# Identification and Validation of Major QTLs, Epistatic Interactions, and Candidate Genes for Soybean Seed Shape and Weight Using Two Related RIL Populations

**DOI:** 10.3389/fgene.2021.666440

**Published:** 2021-05-28

**Authors:** Mahmoud A. Elattar, Benjamin Karikari, Shuguang Li, Shiyu Song, Yongce Cao, Muhammed Aslam, Aiman Hina, Salah Fatouh Abou-Elwafa, Tuanjie Zhao

**Affiliations:** ^1^National Center for Soybean Improvement, Key Laboratory of Biology and Genetic Improvement of Soybean (Ministry of Agriculture), State Key Laboratory of Crop Genetics and Germplasm Enhancement, Nanjing Agricultural University, Nanjing, China; ^2^Agronomy Department, Faculty of Agriculture, Minia University, Minia, Egypt; ^3^Agronomy Department, Faculty of Agriculture, Assiut University, Assiut, Egypt

**Keywords:** *Glycine max*, QTL mapping, QTL clusters, marker assisted breeding, epistatic interactions, candidate genes

## Abstract

Understanding the genetic mechanism underlying seed size, shape, and weight is essential for enhancing soybean cultivars. High-density genetic maps of two recombinant inbred line (RIL) populations, LM6 and ZM6, were evaluated across multiple environments to identify and validate M-QTLs as well as identify candidate genes behind major and stable quantitative trait loci (QTLs). A total of 239 and 43 M-QTLs were mapped by composite interval mapping (CIM) and mixed-model-based composite interval mapping (MCIM) approaches, from which 180 and 18, respectively, are novel QTLs. Twenty-two QTLs including four novel major QTLs were validated in the two RIL populations across multiple environments. Moreover, 18 QTLs showed significant AE effects, and 40 pairwise of the identified QTLs exhibited digenic epistatic effects. Thirty-four QTLs associated with seed flatness index (FI) were identified and reported here for the first time. Seven QTL clusters comprising several QTLs for seed size, shape, and weight on genomic regions of chromosomes 3, 4, 5, 7, 9, 17, and 19 were identified. Gene annotations, gene ontology (GO) enrichment, and RNA-seq analyses of the genomic regions of those seven QTL clusters identified 47 candidate genes for seed-related traits. These genes are highly expressed in seed-related tissues and nodules, which might be deemed as potential candidate genes regulating the seed size, weight, and shape traits in soybean. This study provides detailed information on the genetic basis of the studied traits and candidate genes that could be efficiently implemented by soybean breeders for fine mapping and gene cloning, and for marker-assisted selection (MAS) targeted at improving these traits individually or concurrently.

## Introduction

Soybean (*Glycine max* L. Merr.) is one of the most important food crops, being a rich source of dietary protein (69%) and providing over 50% edible oil globally, and has a significant role in health and biofuel (Hoeck et al., 2003). Besides improving soil fertility by integrating atmospheric nitrogen in the soil through a synergistic interaction with microorganisms, because of its high nutritional value, soybean is used in human food and animal feed (Wang et al., 2019). Throughout the last five decades, soybean production in China slightly increased. To meet domestic demands, China imports almost 80% of its requirements of soybean; therefore, improving soybean production is a major aim of soybean breeders to make the country self-sufficient (Liu et al., 2018). Most plant breeders are targeting yield-related traits to improve soybean production.

Seed size is an essential trait in flowering plants and plays a critical role in adaptation to the environment (Tao et al., 2017). However, these traits are complex quantitative traits regulated by polygenes and strongly influenced by environment and genotype × environment (G × E) interaction, and hence it is more difficult to select for based on phenotype (Yao et al., 2014). All soybean varieties developed in tropical and subtropical countries have small seed size compared to the temperate-region varieties. Besides, seed size, shape, and weight are important seed quality traits with significant influence on seed use (Basra, 1995; Teng et al., 2017; Wu et al., 2018). A positive correlation between seed size/weight and seed yield has been reported in several studies. Seed size/weight revealed a positive association with seed germination capability and vigor, thereby significantly affecting the competitive capability of the seedling for nutrient and water resources and light, hence enhancing stress tolerance (Edwards and Hartwig, 1971; Haig, 2013). Dissecting the genetic factors underlying seed size, shape, and weight and their relationship to the ambient environment is essential for improving soybean yield and quality-related traits. In addition, understanding the additive and additive × environment (AE) effects of quantitative trait loci (QTLs) and their contribution to the phenotypic variations would facilitate the application marker-assisted selection (MAS) because it will prominently lead the breeders in the QTL selection and expectation of the outcomes of MAS (Jannink et al., 2009).

A major aim of utilizing linkage mapping in plant breeding is to deepen our understanding of the inheritance and genetic architecture of quantitative traits and detect markers that can be employed as indirect selection tools in plant breeding (Bernardo, 2008; Abou-Elwafa, 2016a). In this regard, QTL mapping has been regularly used for detecting the QTL/gene underlying the quantitative traits such as seed size, shape, and weight in crop plants. As known, parental diversity and marker density greatly influence the accuracy and precision of QTL mapping. Besides, the population size used in most of the previously published reports for genetic mapping studies usually varied from 50 to 250 individuals; however, larger populations are needed for high-resolution mapping. A high-density genetic map facilitates the detection of closely linked markers associated with QTLs and provides an effective base for investigating quantitative traits (Mohan et al., 1997; Galal et al., 2014; Tewodros and Zelalem, 2016). The statistical difference between phenotypic data obtained from various environments could enhance the accuracy to detect QTL position (Zhao and Xu, 2012). Previous studies identified important seed size and shape QTLs, which were also associated with hundred seed weight (HSW); however, most of the studies used low-density genetic maps based on restriction fragment length polymorphism, simple-sequence repeat markers, and biochemical and morphological markers which have a large confidence interval with a low resolution of QTLs that are not suitable for candidate gene identification (Bernardo, 2008; Han et al., 2012; Abou-Elwafa, 2016a,b). Therefore, it is crucial to employ high-density genetic maps to detect more new recombination in a population, which will increase the accuracy of QTL mapping, candidate gene identification, and MAS (Mahmoud et al., 2018; Cao et al., 2019; Hina et al., 2020). Recent advances in genetic and genomic tools and approaches have facilitated the identification of QTLs associated with various agronomic traits in different crop species, including soybean, and the identification of candidate genes underlying these genomic regions (Peterson et al., 2012; Sun et al., 2012; Xie et al., 2018; Zhang F. et al., 2019; Hina et al., 2020; Kajiya-Kanegae et al., 2020). Although epistatic interaction has a stronger effect on inbreeding depression, heterosis, adaptation, speciation, and reproductive isolation (Ma et al., 2015), previous studies focused mostly on identifying main-effect QTLs associated with seed sizes, shapes, and 100-seed weight in soybean. To date, at least 441, 52, and 297 QTLs for seed size, shape, and HSW have been reported^1^ based on various genetic contexts, advances in marker technology, statistical methods, and multiple environments. However, most of these QTLs are minor (*R*^2^ < 10%), not stable, and with larger genomic regions/confidence intervals (Han et al., 2012; Hu et al., 2013; Kato et al., 2014). Recently, there have been limited studies on detecting QTLs with epistatic effects and their interactions with the environment (QEs) (Panthee et al., 2005; Zhang et al., 2011, 2018; Liang et al., 2016). Knowledge about the molecular mechanisms underlying soybean seed size, shape, and weight is still limited. So far, only two seed sizes/weight-related genes have been cloned and characterized from the soybean, i.e., the *Glyma20g25000 (ln)* gene that has a significant impact on seed size and number of seeds per pod (Jeong et al., 2012) and the *PP2C-1* gene that enhances seed size/weight (Lu et al., 2017). Therefore, it is essential to identify major and stable QTLs and candidate genes related to seed size, shape, and weight to improve our understanding of genetic mechanisms controlling these important traits in soybean (Kato et al., 2014; Zhang et al., 2018). The present study aimed to (i) map main-effect QTLs (M-QTLs), additive × additive (AA) QTLs, and QE for seed size, shape, and weight traits; (ii) employ two QTL mapping approaches to validate the identified QTLs in two mapping populations across multiple environments; (iii) analyze the epistatic QTL pairs and their interactions with the environment for further utilization of these QTLs in soybean genetic improvement; and (iv) mine potential candidate genes for the major (*R*^2^ > 10%) and stable (identified across multiple environments or populations) QTLs. We hypothesize that the results of this study would provide comprehensive knowledge on the genetic bases for these traits and mined candidate genes would serve as a foundation for functional validation and verification of some genes for seed size, shape, and weight in soybean. Besides, the results would be useful for the application of marker-assisted breeding (MAB) in soybean.

## Materials and Methods

### Plant Materials and Experiments

Two recombinant inbred line (RIL) populations, i.e., ZM6 and LM6, comprising 126 and 104 lines, respectively, were used in the present study. The two populations were developed by single seed descent (SSD) from crosses between the genotypes Zhengyang (Z) and Linhefenqingdou (L) as female parents and the M8206 (M6) genotype as the male parent. The two female parents, Z and L, have an average 100-seed weight of 17.1 and 35 g, respectively, whereas the male parent has an average 100-seed weight of 13.7 g.

The two RIL populations along with their parents were evaluated for seed size, shape and HSW across multiple environments. Experiments were conducted in the Jiangpu Experimental Station (33° 030’ N and 63° 118’ E), Nanjing, Jiangsu Province, in the 2012, 2013, 2014, and 2017 growing seasons (designated as 12JP, 13JP, 14JP, and 17JP, respectively), the Fengyang Experimental Station, Chuzhou, Anhui Province (32° 870’ N and 117° 560’ E), in the 2012 growing season (designated as 12FY), and the Yancheng Experimental Station, Yancheng, Jiangsu Province (33° 410’ N and 120° 200’ E), in 2014 (designated as 14YC). Plants were sown in June and harvest was done in October of the same year. Experiments were designed in a randomized complete blocks design (RCBD) with three replications. The experimental plot was one row of 2-m-long at 5-cm plant-to-plant distance and 50-cm row-to-row distance. Planting and post-planting operations were carried out following the recommended agronomic practices.

### Phenotypic Evaluation and Statistical Analysis

**FIGURE 1:**
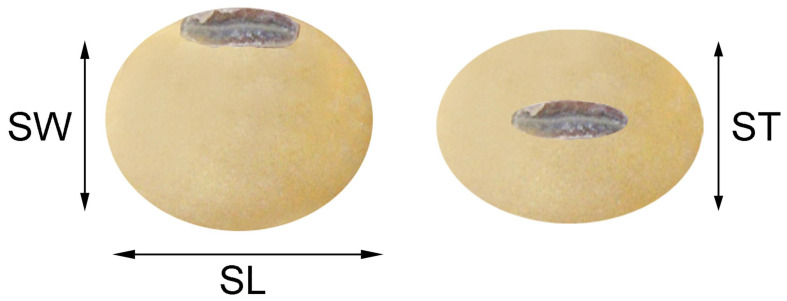
Measuring seed width (SW), length (SL), and thickness (ST).

Eight seed-related traits including seed length (SL), seed width (SW), seed thickness (ST), seed length/width (SLW), seed length/thickness (SLT), seed width/thickness (SWT), flatness index (FI), and 100-seed weight (HSW) were evaluated in LM6 and ZM6 populations under all environments. Phenotypic data were measured and recorded according to standard procedures (Tomooka et al., 2002; Cheng et al., 2006). In brief, seeds harvested from 10 guarded plants in the middle of each row were used for estimating SL, SW, ST, and HSW. The SL was measured as the longest dimension over the seed equivalent to the hilum. SW was measured as the longest dimension across the seed vertical to the hilum. ST was measured as the longest dimension from top to bottom of the seed. The SL, SW, and ST were estimated in millimeters (mm) using the Vernier caliper instrument, according to Kaushik et al. (2007) (Figure 1). Seed shape was identified by calculating three different ratios, i.e., SL/SW (SLW), SL/ST (SLT), and SW/ST (SWT), and FI. The ratios between the SL, SW, and ST were estimated from the individual values of the length, width, and thickness of the seeds according to Omokhafe and Alika (2004), while FI was calculated following the formula elaborated by Cailleux (1945) and Cerdà and Garcıa-Fayos (2002) to describe seed shape:

**FIGURE 2:**
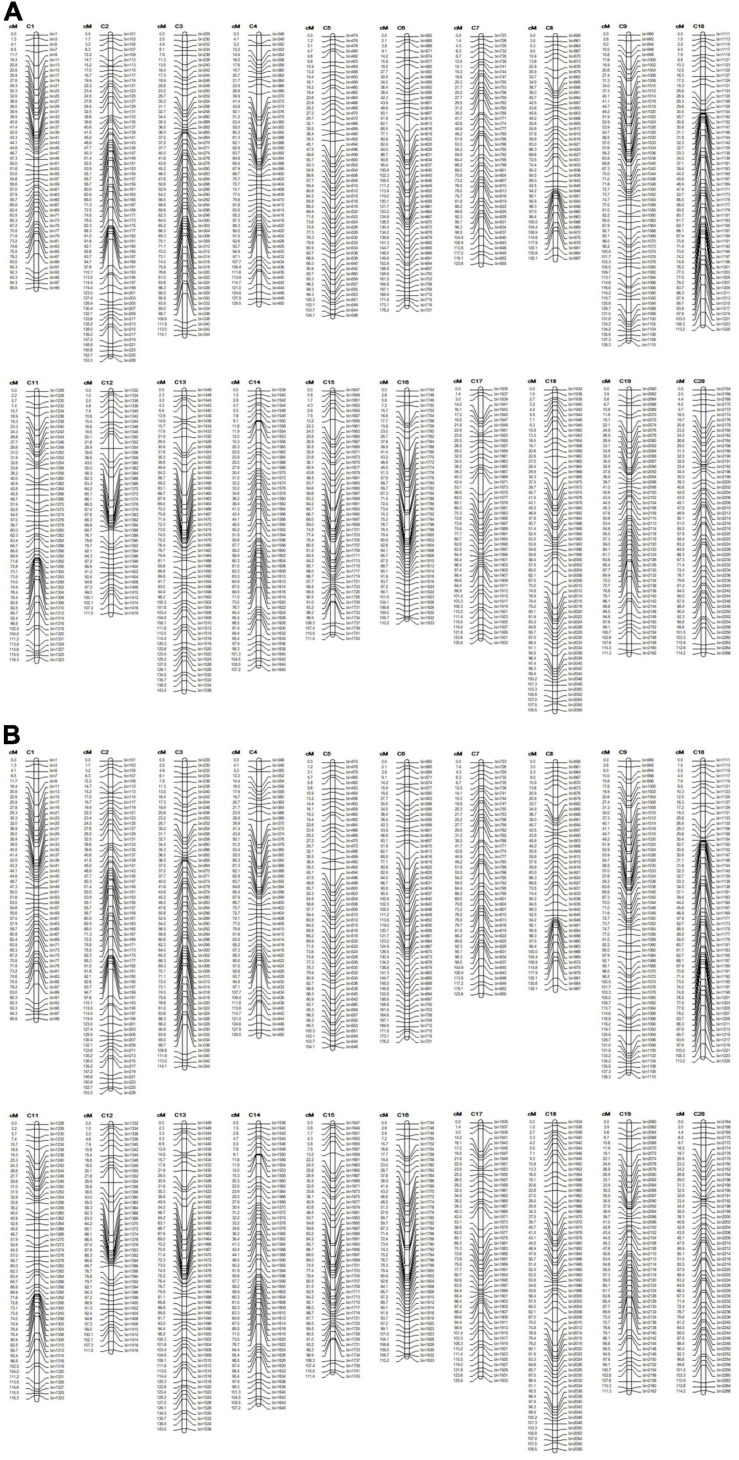
The genetic linkage map of two soybean RIL populations. **(A)** The genetic linkage map for the LM6 population, and **(B)** the genetic linkage map for the ZM6 population. Map distances are shown in centimorgans (cM).

$$F\,I=\frac{\left(L+W\right)}{2\,T}$$

where *L* is the SL, *W* is the SW, and *T* is the ST.

It extended from a value of 1 for the round seeds to more than 2 for skinny seeds. The HSW was expressed as an average of five measurements of 100 randomly selected seeds.

Descriptive statistics of the seed size, seed shape, and HSW traits were calculated using the SPSS software, version 24^2^. Analysis of variance (ANOVA) for each environment and the combined overall environments (CE) was performed using the PROC GLM procedure in SAS software based on the random model (SAS Institute Inc. v. 9.02, 2010, Cary, NC, United States). Broad-sense heritability (*h*^2^) in individual environments was estimated as:

$$h^{2}=\sigma_{g}^{2}/\left(\sigma_{g}^{2}+\sigma_{e}^{2}\right)$$

whereas in the CE *h*^2^ was estimated as follows:

$$h^{2}=\sigma_{g}^{2}/\left(\sigma_{g}^{2}+\sigma_{g\,e}^{2}/n+\sigma_{e}^{2}/n\,r\right)$$

where σ^2^*_*g*_*, σ^2^*_*e*_*, and σ^2^*_*ge*_* are the variance components estimated from the ANOVA for the genotypic, error, and genotype × experiment variances, respectively, with *r* as the number of replicates and *n* as the number of environments. All the parameters were assessed from the expected mean squares in ANOVA. The Pearson correlation coefficient (*r*) between seed size, seed shape, and HSW traits was calculated from the mean data using the SAS PROC CORR with data obtained for CE (average across environments) for each population.

### Construction of Genetic Maps and QTL Analysis

Genetic map information was obtained from the National Center for Soybean Improvement, Nanjing Agricultural University. High-density genetic maps of the ZM6 and LM6 populations comprise 2601 and 2267 bin markers by using the RAD-seq technique, respectively (Supplementary Table 1), which were constructed as previously reported (Karikari et al., 2019; Zhang X. et al., 2019). The total lengths of the ZM6 and LM6 genetic maps were 2630.22 and 2453.79 cM, with an average distance between the markers 1.01 and 1.08 cM, respectively (Supplementary Table 1). The average marker per chromosome was 130 and 113 for the ZM6 and LM6 maps, respectively, with average genetic distances per chromosome of 131.51 and 122.69 cM, respectively (Figure 2 and Supplementary Table 1).

### Mapping of Main- and Epistatic-Effect QTLs

The WinQTLCart 2.5 software (Wang et al., 2006) was employed to identify the M-QTLs using the average values of seed size, seed shape, and 100-seed weight from the individual environments and overall environments with the composite interval mapping model (CIM) (Zeng, 1994). The software running features were 10 cM window size, 1 cM running speed, the logarithm of odds (LOD) (Morton, 1955) threshold which was computed using 1000 permutations because of an experiment-wide error proportion of *P* < 0.05 (Churchill and Doerge, 1994), and the confidence interval which was determined using a 1-LOD support interval, which was controlled by finding the local on the two sides of a QTL top that is compatible with a reduction of the 1 LOD score. QTLs detected within overlapping intervals in different environments were considered the same (Qi et al., 2017). To identify the genetic effects of the QTLs, i.e., additive QTLs, additive × additive (AA), AE, and AA × environment (AAE), the mixed-model-based composite interval mapping (MCIM) procedure was employed in the QTLNetwork V2.1 software (Yang et al., 2008). The critical F-value was calculated by a permutation test with 1000 permutations for MCIM. The effects of QTLs were assessed using the Markov Chain Monte Carlo (MCMC) approach. Epistatic effects, candidate interval selection, and putative QTL detection were estimated with an experiment-wide error proportion of *P* < 0.05 (Yang et al., 2007; Xing et al., 2012).

### *In silico* Identification of Candidate Genes

QTLs identified in two or more environments with an *R*^2^ > 10% were considered as stable and major QTLs (Qi et al., 2017). Genomic regions with several M-QTLs related to different studied traits were identified as a QTL cluster. The Phytozome^3^ and SoyBase (see text footnote 1) online platform repositories were employed to retrieve all model genes within the physical interval position of the QTL clusters. Potential candidate genes were identified based on gene annotations (see text footnotes 1 and 3) and the reported putative function of genes implicated in these traits. Gene ontology (GO) enrichment analysis was performed for the identified candidate genes within each QTL cluster region using AgriGO V2.0^4^ (Tian et al., 2017). Gene classification was then carried out using the Web Gene Ontology (WeGO) Annotation Plotting tool, Version 2.0 (Ye et al., 2006). The publicly available RNA-Seq database on the SoyBase website was used to analyze the expression of the candidate genes in various soybean tissues and developmental stages. A heat map to visualize the fold-change patterns of these candidate genes was constructed using the TBtools_JRE 1.068 software (Chen C. et al., 2020).

## Results

### Phenotypic Variations in RIL Populations in Multiple Environments

**FIGURE 3:**
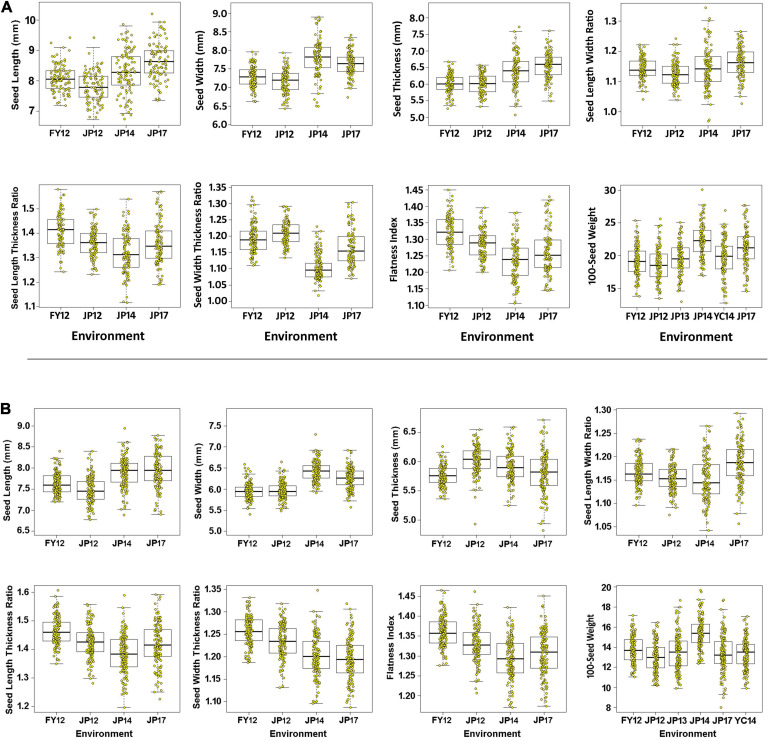
Boxplot for seed size, seed shape, and 100-seed weight traits. The black line in the middle of the box shows the median, the white box indicates the range from the lower quartile to the upper quartile, and the dashed black line and yellow dots represent the dispersion and frequency distribution of the phenotypic data in each of the six environments, i.e., 12FY, 12JP, 13JP, 14JP, 14YC, and 17JP, while **(A,B)** represent LM6 and ZM6 populations.

**FIGURE 4:**
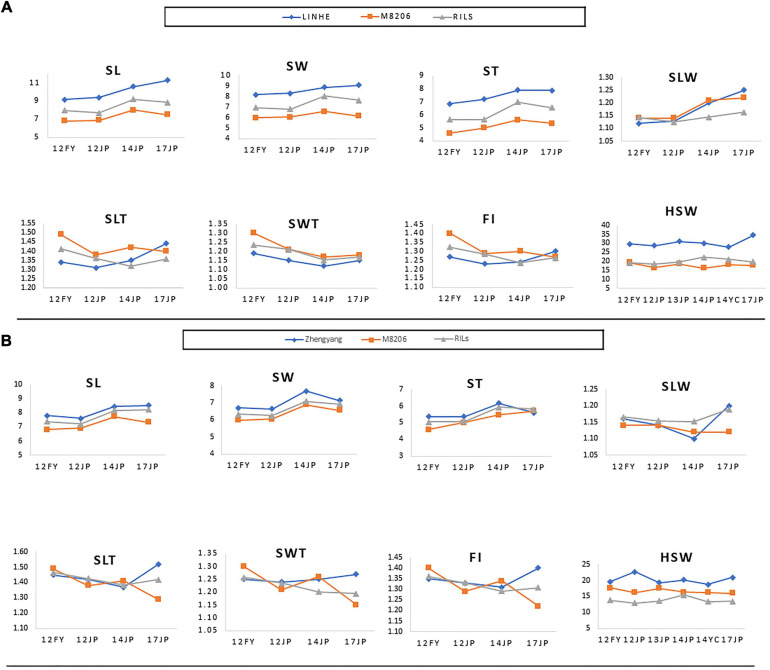
Performance of the parents of the two RIL populations, i.e., LINHE and M8206 **(A)** and Zhengyang and M8206 **(B)** along with the two derived RIL populations, LM6 and ZM6, respectively for seed size and shape traits as well as 100-seed weight among multiple environments. 12JP, 13JP, 14JP, and 17JP indicate phenotyping at the Jiangpu Experimental Station in the 2012, 2013, 2014, and 2017 growing seasons, respectively. 12FY indicates the Fengyang Experimental Station, Chuzhou in the 2012 growing season. 14YC indicates the Yancheng Experimental Station in 2014. SL, seed length (mm); SW, seed width (mm); ST, seed thickness (mm); SLW, seed length to width ratio; SLT, seed length to thickness ratio; SWT, seed width to thickness ratio; FI, flatness index; HSW, 100-seed weight (g).

All measured (SL, ST, SW, and HSW) and calculated (SLW, SLT, SWT, and FI) phenotypic traits exhibited significant differences among the three parental lines across all environments as indicated by the ANOVA (Supplementary Tables 2 and 3). ANOVA revealed that all studied traits were significantly (*P* < 0. 001 or <0.05) influenced by the environment, genotypes, and the genotype × environment interaction (Supplementary Tables 4, 5), indicating the differential response of the genotypes to the changes in environmental cues. The two populations showed continuous phenotypic variations in all studied traits, implying a polygenic inheritance of these traits (Figure 3). The differences in mean phenotypic values among the three parental lines for seed size, seed shape, and HSW traits were constantly high across all studied environments, and their multi-environment means for both populations (Figure 4). Compared to the male parent, the female parent of the LM6 population, Linhefenqingdou, exhibited an average increase of 27.80, 28.19, 31.10, and 41.37% in SL, ST, SW, and HSW, respectively. Meanwhile, in the ZM6 population the female parent Zhengyang surpassed the male parent M8206 by an average of 11.00, 9.66, 7.65, and 17.53% in SL, ST, SW, and HSW across all environments, respectively (Figure 4, Supplementary Tables 2, 3). In both populations, several lines overstep their parents in both directions in all studied traits across all environments, suggesting the occurrence of transgressive segregations within the two populations (Figures 3, 4). The broad-sense heritability (*h*^2^) under individual environments ranged from 66.75 to 98.08%, 64.39 to 95.72%, and 81.45 to 99.36% for seed size, HSW, and seed shape, respectively (Supplementary Tables 2, 3). Meanwhile, *h*^2^ under combined environments (CE) ranged from 78.25 to 87.31%, 65.70 to 90.80%, and 92.95 to 95.72% for seed size, shape, and HSW, respectively. The correlation coefficient (*r*^2^) among SL, ST, and SW exhibited significant positive correlations with each other and with two of the seed shape traits (SLT and SLW) in both populations with *r*^2^ values ranging from 0.79 to 0.91. Meanwhile, SL, ST, and SW exhibited significant negative correlations with the other two seed shape traits (SWT and FI) (Supplementary Table 6). Except for the correlation between SLW and SWT, all the seed shape traits showed significant positive correlations with each other in both populations with *r*^2^ values ranged from 0.33 to 0.95. All seed size traits, i.e., SL, SW, and ST, showed significant positive correlations with HSW with *r*^2^ values ranging from 0.29 to 0.70 in both populations.

### Validation of Identified QTLs Employing Two Mapping Approaches

A total of 92, 99, and 48 M-QTLs associated with seed size, seed shape, and HSW, respectively, were mapped by the CIM approach (Supplementary Tables 7–9). Meanwhile, forty-three QTLs were identified for seed size, shape, and HSW by using MCIM approach (Tables 1, 2). Among these, 22 QTLs were identified and validated by both approaches within the same physical chromosomal position, indicating the dependability and stability of these QTLs. A comparison of the physical chromosomal regions of the QTLs detected by both approaches revealed the identification and validation of four QTLs, i.e., *qSL-7-1_*LM6*_*, *qSW-19-2_*LM6*_*,*qFI-3-1_*LM6*_*, and*qHSW-3-2_*LM6*_*, for the first time in the two populations (LM6 and ZM6) with an *R*^2^ > 10%. Therefore, we considered these QTLs as novel stable and major QTLs that could be used for map-based cloning, candidate gene identification, and QTL stacking into elite cultivars targeted at improving seed size, shape, and HSW in soybean.

**TABLE 1:** 1 QTL naming followed nomenclature q for quantitative trait locus, trait name seed size [seed length (SL), thickness (ST), and width (SW)] and seed shape [length to-thickness (SLT), length-to-width (SLW), width-to-thickness (SWT) ratios, and flatness index (FI)] followed by chromosome number, number of QTLs detected on each chromosome for each trait and subscripted by name of recombinant inbred line population (LM6 and ZM6). 2 Genetic position of QTL (cM). 3 Additive effect (A) and phenotypic variation explained by QTL(PVE). 4 Additive by environment effect in FY2012 (AE1); JP2012 (AE2); JP2014 (AE3); and JP2017 (AE4).

QTL^1^	Marker interval	Position (cM)^2^	Physical position (bp)	Additive – effect (A)^3^		Additive × environment effect (AE)^4^					References
				*A*	*H*^2^%	AE1	AE2	AE3	AE4	*H*^2^%	
*qSL-7-1_*LM6*_*	bin744–bin745	15.25	3,324,836–3,459,470	0.16**	17.45	NS	NS	NS	NS	0.07	Hu et al., 2013
*qSL-13-6_*LM6*_*	bin1535–bin1536	140.15	43,244,220–44,026,619	0.13**	6.08	NS	NS	NS	NS	0.28	Salas et al., 2006
*qSW-13-5_*LM6*_*	bin1536–bin1537	143.04	43,953,331–44,408,971	0.51**	10.52	0.18*	−0.18*	0.16*	0.21**	0.15	Salas et al., 2006
*qSW-19-2_*LM6*_*	bin2100–bin2101	42.57	34,493,194–34,882,495	0.18**	19.47	−0.13**	−0.11*	0.06*	0.09*	0.18	New
*qST-9-5_*LM6*_*	bin1022–bin1023	51.88	7,308,659–7,459,924	0.12**	8.6	NS	NS	NS	NS	0.03	Salas et al., 2006
*qST-18-4_*LM6*_*	bin1979–bin1980	43.69	9,222,099–10,402,370	0.06**	11.34	NS	NS	NS	NS	0.07	Fang et al., 2017
*qSLT-3-1_*LM6*_*	bin247–bin248	19.49	3,119,582–3,515,594	0.12**	2.67	NS	−.22**	NS	NS	4.93	New
*qSLT-14-1_*LM6*_*	bin1586–bin1587	42.38	7,850,227–8,143,522	−0.09**	1.96	NS	0.27**	NS	NS	6.73	Li et al., 2010
*qSLT-17-5_*LM6*_*	bin1883–bin1884	70.26	13,441,932–13,696,232	−0.13**	2.41	NS	NS	NS	NS	1.05	New
*qSWT-7-5_*LM6*_*	bin816–bin817	79.69	29,822,346–35,034,728	−0.67**	12.58	NS	NS	NS	NS	1.27	Fang et al., 2017
*qFI-3-3_*LM6*_*	bin244–bin245	17.30	2,790,829–2,980,527	0.16**	11.48	NS	NS	NS	NS	0.13	New
*qFI-5-1_*LM6*_*	bin476–bin477	0.21	1–5,29,217	0.17**	21.22	NS	NS	NS	NS	0.15	New
*qFI-8-6_*LM6*_*	bin954–bin955	95.88	35,158,414–37,964,850	0.17**	15.74	0.21*	−0.19*	0.14**	0.21*	0.11	New
*qFI-9-5_*LM6*_*	bin1030–bin1031	56.45	20,192,294–27,035,074	−0.13**	4.27	NS	NS	NS	NS	0.21	New
*qFI-11-3_*LM6*_*	bin1290–bin1291	69.93	18,546,688–18,767,705	0.1**	2.27	NS	NS	NS	NS	1.47	New
*qFI-16-1_*LM6*_*	bin1745–bin1746	1.66	6,97,999–9,08,917	−0.08**	14.74	NS	NS	NS	NS	0.11	New
*qSL-1-4_*ZM6*_*	bin4–bin5	3.23	7,54,691–1,375,000	0.05**	3.33	NS	NS	NS	NS	0.08	New
*qSL-9-2_*ZM6*_*	bin1174–bin1175	90.55	3,850,7474–38,736,001	0.04*	2.06	NS	NS	0.07*	NS	2.29	New
*qSL-10-1_*ZM6*_*	bin1236–bin1237	24.62	3,150,454–3,297,961	0.05**	13.67	NS	NS	NS	NS	1.4	New
*qSL-10-2_*ZM6*_*	bin1334–bin1335	106.35	44,226,599–44,378,813	0.05**	5.57	−0.1**	0.06*	0.09**	−0.07*	3.58	Li et al., 2010
*qSL-12-4_*ZM6*_*	bin1553–bin1554	97.99	38,615,116–38,812,896	−0.05**	3.17	NS	NS	NS	NS	0.83	New
*qSL-13-2_*ZM6*_*	bin1612–bin1613	71.65	25,830,321–26,065,585	−0.08**	6.17	NS	NS	NS	NS	1.33	Fang et al., 2017
*qSL-15-5_*ZM6*_*	bin1918–bin1919	85.59	17,503,517–17,963,129	0.14**	3.56	NS	NS	NS	NS	0.08	Salas et al., 2006
*qSW-8-5_*ZM6*_*	bin959–bin960	73.74	11,970,511–12,228,336	0.04**	3	NS	NS	NS	NS	0.65	New
*qST-10-5_*ZM6*_*	bin1334–bin1335	106.35	44,226,599–44,378,813	0.42**	3.99	NS	NS	NS	NS	3.07	Hu et al., 2013
*qST-10-6_*ZM6*_*	bin1336–bin1337	107.17	44,378,814–44,741,960	−0.41**	2.25	NS	NS	0.15**	NS	3.17	New
*qST-13-5_*ZM6*_*	bin1609–bin1610	67.36	24,985,496–25,641,179	−0.41**	4.1	0.8**	−0.6*	−0.7**	0.61*	3.41	New
*qST-14-3_*ZM6*_*	bin1809–bin1810	104.68	47,489,495–47,717,306	0.04**	2.87	NS	NS	0.05**	NS	2.42	New
*qST-20-1_*ZM6*_*	bin2463–bin2464	4.37	6,62,753–1,045,131	−0.06**	3.78	NS	NS	NS	NS	0.71	New
*qSLW-9-4_*ZM6*_*	bin1172–bin1173	89.06	38,139,739–38,507,473	0.99**	2.51	NS	NS	NS	NS	1.25	Li et al., 2010
*qSLW-10-2_*ZM6*_*	bin1275–bin1279	60.40	14,218,565–17,808,941	0.82**	12.74	NS	−0.92*	0.95*	NS	2.3	New
*qSLW-13-5_*ZM6*_*	bin1653–bin1654	102.63	32,704,220–33,303,066	−0.1**	1.15	NS	0.3**	NS	NS	3.95	Salas et al., 2006
*qSLT-5-3_*ZM6*_*	bin600–bin599	93.89	40,328,493–40,882,874	0.013**	11.18	NS	NS	NS	NS	0.32	Salas et al., 2006
*qFI-17-6_*ZM6*_*	bin2177–bin2178	130.75	41,009,636–41,399,912	0.06**	14.81	NS	NS	NS	NS	0.6	New
*qFI-20-1_*ZM6*_*	bin2461–bin2462	2.80	1–6,62,752	0.08**	5.45	NS	NS	0.12*	0.09*	0.28	New

*^∗^*p* < 0.05; ^∗∗^*p* < 0.01; NS, non-significant. A indicates additive effects, those with positive values show beneficial alleles from parents Zhengyang and Linhefenqingdou, while those with negative values show beneficial alleles from parent Meng 8206.*

**TABLE 2:** 1 QTL naming followed nomenclature q for quantitative trait locus, trait name 100-seed weight (HSW) followed by chromosome number, number of QTLs detected on each chromosome for each trait and subscripted by name of recombinant inbred line population (LM6 and ZM6). 2 Genetic position of QTL (cM). 3 Additive effect (A) and phenotypic variation explained by QTL (PVE). 4 Additive by environment effect in FY2012 (AE1); JP2012 (AE2); JP2013 (AE3); JP2014 (AE4); YC2014 (AE5); and JP2017 (AE6).

QTL^1^	Marker interval	Position (cM)^2^	Physical position (bp)	Additive – effect (A)^3^		Additive × environment effect (AE)^4^							References
				*A*	*H*^2^%	AE1	AE2	AE3	AE4	AE5	AE6	*H*^2^%	
*qHSW-3-2_*LM6*_*	bin255–bin256	30.64	5,83,3775–6,78,0840	0.61**	10.75	NS	NS	NS	NS	NS	NS	0.16	Li et al. (2008)
*qHSW-14-3_*LM6*_*	bin1640–bin1641	101.33	48,267,526–48,523,627	0.48**	0.28	0.52*	NS	NS	NS	−0.16*	NS	1.99	New
*qHSW-8-3_*ZM6*_*	bin963–bin964	80.64	12,871,276–13,803,222	0.29**	3.21	NS	NS	NS	0.34*	−0.41*	NS	0.42	Han et al., 2012
*qHSW-9-1_*ZM6*_*	bin1162–bin1163	77.51	35,758,796–36,561,550	0.40**	2.47	NS	NS	NS	NS	NS	NS	0.88	New
*qHSW-13-3_*ZM6*_*	bin1611–bin1612	69.99	25,641,180–26,012,595	−0.33**	5.7	NS	NS	−0.38*	NS	NS	NS	1.13	Funatsuki et al. (2005)
*qHSW-14-2_*ZM6*_*	bin1746–bin1747	28.67	4,176,245–4,861,311	−0.15**	1.66	NS	NS	NS	NS	NS	NS	0.17	New
*qHSW-14-4_*ZM6*_*	bin1809–bin1810	104.68	47,489,495–47,717,306	0.46**	2.78	0.54**	NS	NS	0.43*	NS	−0.69**	4.75	New
*qHSW-16-3_*ZM6*_*	bin2043–bin2044	103.81	35,441,262–35,607,069	0.21**	1.67	NS	NS	NS	NS	NS	NS	0.29	New

*^∗^*p* < 0.05; ^∗∗^*p* < 0.01; NS, non-significant. A indicates additive effects, those with positive values show beneficial alleles from parents Zhengyang and Linhefenqingdou, while those with negative values show beneficial alleles from parent Meng 8206.*

### Identification of the Main Effects of the Stable Additive Seed Size QTLs

**FIGURE 5:**
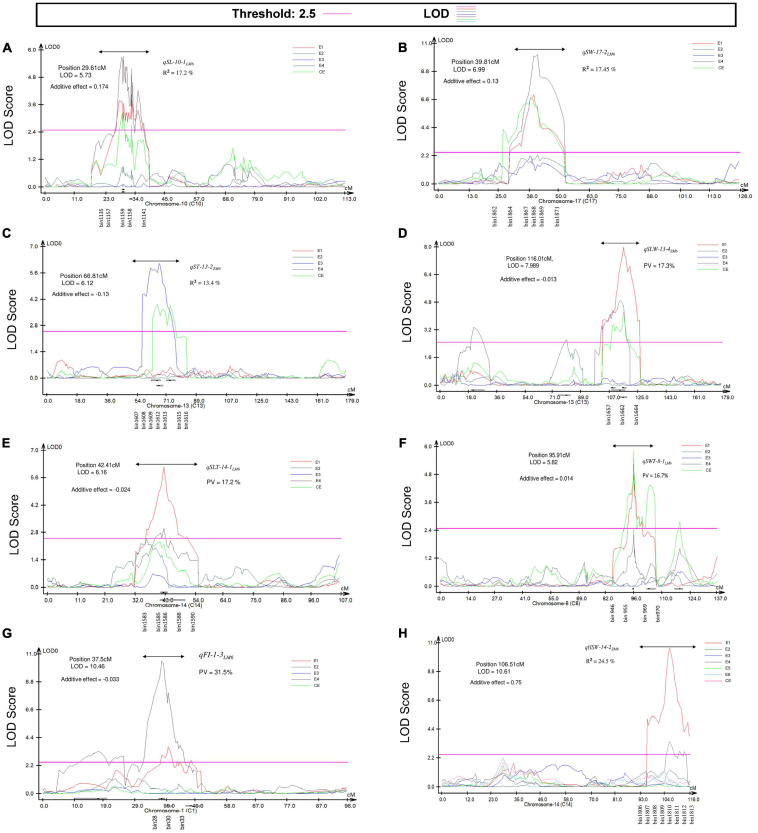
Position of the most prominent QTL detected by the CIM approach associated with seed size and seed shape traits in the LM6 and ZM6 RIL populations grown in multiple environments indicated as with E1, FY2012; E2, JP2012; E3, JP2013; E4, JP2014; E5, YC2014; E6, JP2017, respectively, in addition to the combined environment (CE). **(A)** LOD curve for *qSL-10-1_*LM6*_*, **(B)** LOD curve for *qSW-17-2_*LM6*_*, **(C)** LOD curve for *qST-13-2_*ZM6*_*, **(D)** LOD curve for *qSLW-13-4_*ZM6*_*, **(E)** LOD curve for *qSLT-14-1_*LM6*_*, **(F)** LOD curve for *qSWT-8-1_*LM6*_*, **(G)** LOD curve for *qFI-1-3_*LM6*_*, and **(H)** LOD curve for *qHSW-14-2_*ZM6*_*. The LOD threshold (2.5) is indicated by a pink line. The double-headed arrow denotes the location of prominent QTL. The *X* and *Y*-axes represent chromosome and LOD score, respectively.

A total of 92 M-QTLs were mapped for seed size-related traits, i.e., SL, SW, and ST, on all soybean chromosomes, except chromosomes 1 and 12, with LOD scores and *R*^2^ values ranging from 2.5 to 10.3 and 5.0 to 19.7%, respectively, in the two populations (Supplementary Table 7 and Supplementary Figures 1a–c). Out of these, 30 M-QTLs for SL, 35 for SW, and 27 for ST with alleles underlying QTLs inherited from either of the parents. Seventy-two M-QTLs were mapped in one environment while the remaining 20 were mapped within overlapping regions in at least one environment with or without CE. Forty-seven QTLs exhibiting *R*^2^ > 10% were considered as major QTLs. The most prominent QTL was the *qSW-17-2_*LM6*_* (LOD = 6.70–10.29, and *R*^2^ = 16.60–18.30%), which was detected within the physical position 6,844,412–9,645,325 bp in 14JP and CE (Figure 5b). Likewise, the *qSL-10-2_*ZM6, LM6*_* (LOD = 6.08–6.89, and *R*^2^ = 15.4–17.1% in ZM6 (17JP) and LM6 (14JP) populations) was located to the physical position between 41,454,163 and 43,944,243 bp.

### Main Effects of the Stable Additive Seed Shape QTLs

In total, 99 M-QTLs related to seed shape traits (SLT, SLW, SWT, and FI) were mapped to 19 soybean chromosomes excluding chromosome 2 in both populations across four environments and the CE with LOD scores of 2.50–10.44 and *R*^2^ of 5.12–31.56% by the CIM approach (Supplementary Table 8 and Supplementary Figures 1d–g). From the 99 M-QTLs, 22, 33, 11, and 22 were detected for SLT, SLW, SWT, and FI, respectively (Supplementary Table 8). Among them, 71 M-QTLs were detected in multiple environments, while 28 were mapped in at least one environment either with or without the CE. Eight M-QTLs for SLW (*qSLW-3-2_*LM6*_*, *qSLW-5-3_*ZM6, LM6*_*, *qSLW-9-3_*LM6, ZM6*_*, *qSLW-13-4_*ZM6*_, _*LM6*_*, *qSLW-15-1_*LM6*_, qSLW-15-3_*LM6*_*, *qSLW-16-2_*ZM6*_*, and *qSLW-16-3_*ZM6*_*) were mapped in at least one environment with or without the CE. Similarly, seven M-QTLs for SLT (*qSLT-1-3_*LM6*_*, *qSLT-5-3_*ZM6*_*, *qSLT-11-1_*LM6*_*, *qSLT-13-1_*LM6*_*, *qSLT-14-1_*LM6*_, qSLT-16-1_*LM6*_*, and *qSLT-20-1_*ZM6*_*) were mapped in at least one environment with or without the CE (Supplementary Table 8 and Supplementary Figure 1e). Likewise, four M-QTLs (*qSWT-8-1_*LM6*_*, *qSWT-11-2_*ZM6, LM6*_*, *qSWT-13-1_*ZM6*_*, and *qSWT-17-1_*ZM6*_*) were mapped for SWT in at least one environment with or without the CE (Supplementary Table 8 and Supplementary Figure 1f). Also, a total of 10 M-QTLs (*qFI-1-1_*ZM6*_*, *qFI-1-2_*ZM6, LM6*_*, *qFI-1-3_*LM6*_*, *qFI-1-4_*ZM6, LM6*_*, *qFI-3-1_*LM6*_*, *qFI-3-3_*ZM6*_*, *qFI-5-2_*ZM6*_*, *qFI-11-1_*LM6*_*, *qFI-14-1_*LM6*_*, *qFI-17-1_*ZM6*_*, and *qFI-20-1_*ZM6*_*) were considered as stable QTLs as they were detected in multiple environments. Several physical regions which harbored at least two seed shape-related traits were identified, e.g., the chromosomal region between 1,730,667 and 3,014,518 bp on chr01 harbors *qSLT-1-2_*ZM6*_*, *qSWT-1-1_*ZM6*_*, and *qFI-1-2_*ZM6, LM6*_*, and the chromosomal region between 4,946,300 and 35,955,471 bp on chr01 which comprises *qSLT-1-3_*LM6*_*, *qSWT-1-2_*LM6*_*, and *qFI-1-3_*LM6*_* (Supplementary Figure 2 and Supplementary Table 8). Two M-QTLs (*qSLW-3-2_*LM6*_* and *qFI-3-1_*LM6*_*) located to the physical region between 1,509,548 and 3,515,594 bp on chr03. The QTLs *qSLT-5-3_*ZM6*_*, *qFI-5-2_*ZM6*_*, and *qSLW-5-3_*ZM6, LM6*_* reside the physical region between 38,035,798 and 41,186,985 bp on chr05 (Supplementary Figure 2 and Supplementary Table 8). The *qSLT-11-1_*LM6*_* and *qFI-11-1_*LM6*_*, *qSLT-13-1_*LM6*_*, *qSLW-13-4_*ZM6, LM6*_* and *qSWT-13-1_*ZM6*_*, and *qFI-14-1_*LM6*_, qSLT-14-1_*LM6*_* and *qFI-14-1_*LM6*_* were located to the physical regions of 17,145,381–23,469,672, 33,303,067–39,562,563, and 3,468,251–8,668,367 bp on chr11, chr13, and chr14, respectively (Supplementary Figure 2 and Supplementary Table 8). The *qSLW-16-2_*ZM6*_*, *qSLT-16-1_*LM6*_* and *qSLW-16-3_*ZM6*_*, *qFI-17-1_*ZM6*_* and *qSWT-17-1_*ZM6*_*, and *qSLT-20-1_*ZM6*_* and *qFI-20-*1_*ZM6*_ were located to the chromosomal regions of 26,903,205–31,959,397 on Chr16, 40,207,655–41,672,092 bp on Chr17, and 1–1,115,156 bp on Chr20, respectively (Supplementary Figure 2 and Supplementary Table 8).

### Main Effects of the Stable Additive Seed Weight QTLs

A total of 48 M-QTLs for HSW were detected, from which 27 were detected in a specific environment and 21 were mapped in at least one environment with or without the CE (Supplementary Table 9 and Supplementary Figure 1h). The LOD scores and *R*^2^ values of these M-QTLs ranged from 2.51 to 10.61 and 4.8 to 24.5%, respectively. The highest number of M-QTLs (six QTLs) was mapped on Chr 04 followed by Chr10 with five M-QTLs, and the lowest number of one M-QTL was mapped to Chr 02, 09, 17, and 18. The most prominent M-QTLs were *qHSW-14-2_*ZM6*_*, *qHSW-10-3_*LM6*_*, and *qHSW-10-4_*LM6*_* with LOD scores and *R*^2^ values of 10.61 and 24.50% (Figure 5), 7.57 and 17.60%, and 7.20 and 16.90%, respectively. Among those 21 M-QTLs, *qHSW-4-3_*LM6*_*, *_*ZM6*_, qHSW-6-2_*LM6*_*, *qHSW-10-1_*LM6*_*, *qHSW-13-1_*ZM6*_*, *qHSW-15-2_*LM6*_*, and *qHSW-15-4_*LM6*_* were mapped in at least three environments with an average *R*^2^ of 13.01%.

### Analysis of Additive-Effect QTLs and Additive × Environment QTL Interactions

The mixed-MCIM approach implemented in the QTL Network V2.1 software for both RIL populations across multiple environments identified 35 *AA* QTLs on 17 chromosomes related to seven seed size and seed shape traits. These comprise 9, 3, 7, 3, 4, 1, and 8 *A* QTLs associated with SL, SW, ST, SLW, SLT, SWT, and FI, respectively, in the LM6 and ZM6 populations across all environments (Table 1). The contributed allele of 11 QTLs of them which reduces seed size and seed shape values through significant additive effects is inherited from the M8206 parent. Meanwhile, the contributed allele of the remaining 24 QTLs, which enhances seed size and shape values through significant additive effects, is inherited from either Zhengyang or Linhefenqingdou parent of the ZM6 or LM6 population, respectively (Table 1). Thirteen out of 35 QTLs revealed significant *AE* effects in at least one environment. However, five QTLs, i.e., *qSW-13-5_*LM6*_*, *qSW-19-2_*LM6*_*, *qFI-8-6_*LM6*_*, *qSL-10-2_*ZM6*_*, and *qST-13-5_*ZM6*_*, showed significant or highly significant *AE* among all studied environments (Table 1). The influence of *AE* effects on seed size and seed shape values was environmentally dependent (Table 1). Eight *AA* QTLs associated with HSW were identified on six chromosomes, i.e., Chr 03, 08, 09, 13, 14, and 16, in LM6 and ZM6 populations across six environments (Table 2). Six of those eight QTLs displayed a positive additive effect with the beneficial allele that could increase HSW which is inherited from the female parents (Linhefenqingdou or Zhengyang). Meanwhile, the remaining two QTLs, i.e., *qHSW-13-3_*ZM6*_* and *qHSW-14-2_*ZM6*_*, revealed negative additive effects with the allele that reduces HSW which is inherited from the common male parent (Meng8206) (Table 2). Two QTLs associated with HSW, i.e., *qHSW-14-3_*LM6*_* and *qHSW-8-3_*ZM6*_*, displayed significant *AE* effects in two environments, whereas *qHSW-13-3_*ZM6*_* showed a significant *AE* only in one environment (the 13JP environment). The *qHSW-14-4_*ZM6*_* QTL revealed significant *AE* effects across three different environments, i.e., 12FY, 12JP, and 17JP (Table 2).

### Validation of QTLs and Identification of QTL Clusters

A chromosomal region comprising several identified M-QTLs for different studied seed phenotypic traits was designated as a QTL cluster. Accordingly, 24 QTL clusters on 17 chromosomes with the exception to Chr 02, 12, and 18 were identified (Supplementary Table 10 and Supplementary Figure 2). Among the identified 24 clusters, seven clusters harbored QTLs related to seed size, seed shape, and HSW, five clusters harbored QTLs related only to seed size and seed shape traits, nine clusters comprised QTLs related to seed size and HSW traits, and three clusters harbored QTLs for only seed shape traits (Supplementary Table 10). QTLs within 15 clusters revealed positive additive effects with the beneficial alleles which are inherited from the big seed size and heavy seed weight parents (Zhengyang or Linhefenqingdou). Seven out of 24 clusters contain QTLs that have been detected and validated in the low RIL populations (Supplementary Table 10). The most prominent M-QTL (*qFI-1-3_*LM6*_*) with a LOD score of 3.71–10.44 and *R*^2^ (10.45–31.50%) was located to Cluster-01. Each cluster comprised a different number of QTLs, with the highest number of QTLs, i.e., seven, associated with seed size, shape, and HSW traits which were in cluster-03 at the physical position of 1,509,548–6,780,840 bp allocated as two QTLs related to seed size (*qSL-3-1_*LM6*_* and *qSL-3-2_*LM6*_*), four QTLs for seed shape (*qSLW-3-2 _*LM6*_*, *qFI-3-1_*LM6*_*, *qSLT-3-1_*LM6*_*, and *qFI-3-2_*LM6*_*), and one QTL HSW (*qHSW-3-1_*LM6*_*). Except for *qHSW-3-1_*LM6*_*, all QTLs in this cluster were major QTLs (with an *R*^2^ > 10%). Each of the clusters-13, 16.2, and 17.1 comprises five to six M-QTLs related to seed size and HSW traits identified in one of the two RIL populations and displayed *R*^2^ values of 8.85–13.43, 5.96–11.26, and 6.8–18.30%, respectively (Supplementary Figure 2 and Supplementary Table 10). Another rich region of QTLs was cluster-20 on chr20 that harbors five seed size and shape M-QTLs, i.e., *qFI-20-1_*ZM6*_*, *qSLT-20-1_*ZM6*_*, *qSLW-20-1_*ZM6*_*, *qST-20-1_*ZM6*_*, and *qSW-20-1_*ZM6*_*, from which three are major QTLs with R^2^ of 11.2–19.2% within a physical region of 1.2 Mb (Supplementary Table 10). Cluster-09 comprises five stable (identified in the two populations) seed size, shape, and HSW QTLs with R^2^ values ranging from 12.5 to 16.3%. Cluster-14.1 comprises four major M-QTLs in both populations with *R*^2^ values ranging from 10.4 to 18.4% within the chromosomal region between 5,834,015 and 9,844,637 bp, one from which *qSW-14-2_*ZM6*_* is associated with seed size traits, whereas the other three (*qSLW-14-1_*LM6*_*, *qFI-14-1_*LM6*_*, and *qSLT-14-1_*LM6*_*) were associated with seed shape traits (Supplementary Table 10). Six clusters harboring four M-QTLs each were identified, from which four clusters, i.e., cluster-07, cluster-19.1, cluster-08, and cluster-14.2, comprise QTLs associated with HSW, seed size, and shape trait cluster-10.2 that comprises M-QTLs for seed size and shape traits, and cluster-16.1 that comprises only M-QTLs related to seed shape traits (Supplementary Table 10). The remaining nine clusters have three QTLs each; out of them, cluster-01 and cluster-17.2 comprise major QTLs related only to seed shape traits. Meanwhile, cluster-04.1 and cluster-19.2 contain minor M-QTLs associated with SW, SL, and HSW. Another two clusters comprise M-QTLs for both seed size and shape traits (Supplementary Table 10). The other three M-QTL clusters, i.e., cluster-10.1, cluster-11, and cluster-15, comprise both major and minor QTLs for seed size traits and HSW. Cluster-04.2 comprises the two QTLs *qHSW-4-3_*LM6, ZM6*_* and *qSL-4-1_*ZM6*_* with *R*^2^ values of 13.1–17.7%.

### Analysis of Epistatic-Effect QTLs and Epistatic × Environment QTL Interactions

Analysis of the seed size and shape trait data under all environments identified 38 pairwise epistatic effects (AA) QTLs, from which 2, 13, 6, 2, 3, 5, and 7 pairs were related to SL, SW, ST, SLW, SLT, SWT, and FI traits, respectively, with *R*^2^ values ranging 0.51–11.35% (Table 3). All QTL pairs displayed a high significant *AA* effect. Further analyses revealed that 20 AA QTLs showed significant or highly significant pairwise additive–additive–environment (*AAE*) interaction effects in at least one environment with *R*^2^ values ranging from 0.13 to 5.31% (Table 3). Ten pairs showed significant *AAE* in two environments, i.e., 12FY (AAE1) and 12JP (AAE2), while three pairs displayed significant *AAE* in 12JP (AAE2) and 14JP (AAE3) environments (Table 3). This shows the effect of the environment on gene expression on phenotype development through epistatic effects. Out of the 38 QTLs, 16 pairwise interactions exhibited negative epistatic effects (*AA*) that decrease the values of seed size and shape traits, whereas 22 pairwise interactions exhibited positive epistatic effects (*AA*) that increase the values of seed size and shape traits (Table 3). The pairwise interaction between *qFI-1-1_*ZM6*_* and *qFI-7-3_*ZM6*_* revealed the strongest positive epistatic effect (0.65), whereas the pairwise *qSLT-6-1_*LM6*_* and *qSLW-9-1_*LM6*_* revealed the weakest positive epistatic effect (0.02). Conversely, *qSWT-3-1_*LM6*_* and *qSWT-13-1_*LM6*_* resulted in the strongest negative epistatic effect (−0.71), whereas the *qSLW-2-6_*ZM6*_* and *qSLW-18-3_*ZM6*_* pairwise resulted in the weakest negative epistatic effect (-0.02) (Table 3). Two digenic positive pairwise epistatic QTLs for HSW with highly significant additive × additive (*AA*) effects were identified on four chromosomes (Table 4). The first pairwise is composed of two QTLs, *qHSW-11-1_*LM6*_* and *qHSW-20-1_*LM6*_*, with an *R*^2^ of 3.46%, whereas the second pairwise comprises the two QTLs *qHSW-9-1_*ZM6*_* and *qHSW-16-3_*ZM6*_* with an *R*^2^ of 1.38%. However, the two pairs did not show any significant *AAE* interaction effects across all six environments (Table 4).

**TABLE 3:** Estimated epistatic effects (AA) and environmental (AAE) interaction of QTLs for soybean seed size traits (SL, SW, and ST) and seed shape (SLW, SLT, SWT, and FI) traits across all environments.

RIL	Trait	QTL_i	Chr_i	Interval_i	Pos_i	QTL_j	Chr_j	Interval_j	Pos_j	Epistasis – effect (AA)		Epistasis × environment effect (AAE)				
										AA	*H*^2^%	AAE1	AAE2	AAE3	AAE4	*H*^2^%
LM6	SW	*qSW-2-2_*LM6*_*	2	bin124–bin125	23.92	*qSW-16-2_*LM6*_*	16	bin1757–bin1758	18.19	0.08**	7.24	NS	NS	NS	NS	1.11
		*qSW-16-2_*LM6*_*	3	bin229–bin230	1.96	*qSW-13-1_*LM6*_*	13	bin1509–bin1510	107.72	−0.1**	5.40	NS	NS	NS	NS	0.24
		*qSW-4-2_*LM6*_*	4	bin353–bin354	13.86	*qSW-15-3_*LM6*_*	15	bin1738–bin1739	106.86	−0.53**	2.85	NS	NS	NS	NS	2.34
		*qSW-4-2_*LM6*_*	4	bin353–bin354	13.86	*qSW-15-4_*LM6*_*	15	bin1740–bin1741	110.00	0.33**	7.30	0.11**	−0.11**	NS	NS	3.95
		*qSW-5-1_*LM6*_*	5	bin525–bin526	74.76	*qSW-12-1_*LM6*_*	12	bin1352–bin1353	21.59	−0.11**	3.96	NS	NS	NS	NS	0.96
		*qSW-7-1_*LM6*_*	7	bin784–bin785	53.84	*qSW-15-4_*LM6*_*	15	bin1740–bin1741	110.00	−0.10**	5.83	NS	NS	NS	NS	0.05
		*qSW-8-1_*LM6*_*	8	bin984–bin985	130.62	*qSW-10-3_*LM6*_*	10	bin1219–bin1220	97.61	−0.21**	3.20	−0.13*	0.12*	NS	NS	1.39
		*qSW-10-1_*LM6*_*	10	bin1184–bin1185	62.20	*qSW-20-1_*LM6*_*	20	bin2231–bin2232	69.64	−0.09**	2.20	NS	NS	NS	NS	1.19
		*qSW-10-3_*LM6*_*	10	bin1219–bin1220	97.61	*qSW-16-4_*LM6*_*	16	bin1810–bin1811	87.22	0.14**	1.47	NS	NS	NS	NS	0.18
		*qSW-11-1_*LM6*_*	11	bin1291–bin1292	70.56	*qSW-15-1_*LM6*_*	15	bin1715–bin1716	85.15	−0.17**	4.79	NS	NS	NS	NS	1.08
		*qSW-11-2_*LM6*_*	11	bin1292–bin1296	71.58	*qSW-15-2_*LM6*_*	15	bin1717–bin1718	85.58	0.23**	9.22	NS	NS	NS	0.10**	1.48
	ST	*qST-3-1_*LM6*_*	3	bin237–bin238	8.12	*qST-3-3_*LM6*_*	3	bin344–bin345	114.06	0.05**	0.51	NS	NS	NS	NS	0.56
		*qST-6-3_*LM6*_*	6	bin647–bin648	109.3	*qST-11-1_*LM6*_*	11	bin1274–bin1275	54.45	0.1**	5.55	NS	NS	NS	0.12**	1.40
		*qST-7-2_*LM6*_*	7	bin749–bin750	19.81	*qST-16-3_*LM6*_*	16	bin1755–bin1756	17.12	0.09**	6.31	NS	NS	NS	NS	0.57
		*qST-7-3_*LM6*_*	7	bin783–bin784	53.32	*qST-15-2_*LM6*_*	15	bin1741–bin1742	110.5	−0.12**	5.74	NS	NS	NS	NS	0.69
		*qST-16-1_*LM6*_*	16	bin1744–bin1745	1.66	*qST-17-2_*LM6*_*	17	bin1886–bin1887	72.87	−0.14**	9.36	0.06*	−0.06*	NS	NS	2.04
	SLW	*qSLW-8-2_*LM6*_*	8	bin941–bin942	86.24	*qST-14-3_*LM6*_*	14	bin1625–bin1626	84.91	0.03**	11.35	NS	NS	NS	NS	1.41
	SLT	*qSLT-5-3_*LM6*_*	5	bin560–bin543	92.74	*qSLW-6-2_*LM6*_*	6	bin625–bin626	82.38	−0.04**	5.40	0.03*	NS	NS	NS	2.85
		*qSLT-6-1_*LM6*_*	6	bin584–bin585	28.72	*qSLW-9-1_*LM6*_*	9	bin1095–bin1096	128.66	0.02**	1.61	−0.03*	0.03*	NS	NS	3.00
	SWT	*qSWT-3-1_*LM6*_*	3	bin236–bin237	7.79	*qSWT-13-1_*LM6*_*	13	bin1435–bin1434	15.23	−0.71**	5.19	0.81**	−0.8**	NS	NS	4.57
		*qSWT-6-1_*LM6*_*	6	bin588–bin589	33.52	*qSWT-18-1_*LM6*_*	18	bin2036–bin2037	95.49	−0.62**	3.88	−0.64*	0.63*	NS	NS	0.67
		*qSWT-11-1_*LM6*_*	11	bin1262–bin1263	40.91	*qSWT-20-1_*LM6*_*	20	bin2177–bin2178	19.84	0.14**	8.03	−0.11**	0.15**	NS	NS	4.19
		*qSWT-16-1_*LM6*_*	16	bin1744–bin1745	1.66	*qSWT-17-3_*LM6*_*	17	bin1886–bin1887	72.87	0.11**	11.08	−0.15**	0.17**	NS	NS	5.31
	FI	*qFI-1-5_*LM6*_*	1	bin59–bin60	55.61	*qFI-14-4_*LM6*_*	14	bin1627–bin1628	85.96	0.17**	2.89	NS	NS	0.09*	NS	2.24
		*qFI-5-2LM6*	5	bin516–bin517	65.79	*qFI-10-2_*LM6*_*	10	bin1223–bin1224	108.26	0.04**	10.02	NS	NS	NS	NS	0.02
		*qFI-16-2_*LM6*_*	16	bin1745–bin1746	1.66	*qSWT-17-2_*LM6*_*	17	bin1886–bin1887	38.76	0.02**	6.76	−0.18*	0.02*	NS	0.03*	2.75
	SL	*qSL-12-4_*ZM6*_*	12	bin1553–bin1554	97.99	*qSL-15-5_*ZM6*_*	15	bin1919–bin1920	85.99	−0.06**	1.88	NS	NS	NS	NS	0.13
		*qSL-2-3_*ZM6*_*	2	bin214–bin211	96.57	*qSL-8-6_*ZM6*_*	8	bin1084–bin1085	186.90	0.2**	8.61	NS	NS	NS	NS	0.10
ZM6	SW	*qSW-4-4_*ZM6*_*	4	bin434–bin435	62.42	*qSW-20-5_*ZM6*_*	20	bin2590–bin2591	97.79	−0.12**	6.68	NS	NS	NS	0.07*	0.33
		*qSW-6-3_*ZM6*_*	6	bin684–bin685	86.36	*qSW-6-5_*ZM6*_*	6	bin703–bin704	109.84	0.05**	0.66	−0.08**	0.08**	NS	NS	3.35
	ST	*qST-10-5_*ZM6*_*	10	bin1334–bin1335	106.35	*qST-10-6_*ZM6*_*	10	bin1336–bin1337	107.17	−0.7**	2.36	NS	NS	NS	NS	0.48
	SLW	*qSLW-2-6_*ZM6*_*	2	bin260–bin261	158.24	*qSLW-18-3_*ZM6*_*	18	bin2336–bin2337	123.90	−0.02**	1.14	NS	0.04**	−0.03**	NS	3.74
	SLT	*qSLT-1-4_*ZM6*_*	1	bin53–bin54	43.95	*qSLT-7-2_*ZM6*_*	7	bin884–bin885	103.56	0.03**	4.22	NS	−0.03*	0.03**	NS	2.58
	SWT	*qSWT-1-3_*ZM6*_*	1	bin62–bin63	47.73	*qSWT-8-1_*ZM6*_*	8	bin914–bin915	15.98	0.13**	5.30	NS	NS	NS	NS	0.29
	FI	*qFI-17-6_*ZM6*_*	17	bin2177–bin2178	130.75	*qFI-20-1_*ZM6*_*	20	bin2461–bin2462	2.79	0.51**	1.75	NS	NS	NS	NS	1.20
		*qFI-1-1_*ZM6*_*	1	bin58–bin59	46.05	*qFI-7-3_*ZM6*_*	7	bin884–bin885	103.56	0.65**	5.14	NS	NS	1.01**	NS	1.26
		*qFI-1-3_*ZM6*_*	1	bin72–bin73	66.36	*qFI-7-1_*ZM6*_*	7	bin872–bin873	96.62	0.3**	6.01	NS	NS	NS	NS	0.65
		*qFI-3-1_*ZM6*_*	3	bin289–bin290	27.39	*qFI-18-2_*ZM6*_*	18	bin2313–bin2314	112.03	0.12**	7.29	NS	0.9*	−0.86*	NS	1.88

Chr_i and Chr_j indicate the two sites involved in epistatic interactions; Pos indicates genetic position for each of the sites. *p < 0.05; **p < 0.01; NS, non-significant. AA indicates epistatic effects between two QTLs; those with positive values showing two-locus genotypes being the same as those in parent Linhefenqingdou and Zhengyang (or Meng 8206) have the beneficial effects, while the two-locus recombinants take the negative effects. The case of negative values is the opposite. *H*^2^ indicates phenotypic variation explained by epistatic effects. AE1, FY2012; AE2, JP2012; AE3, JP2014; and AE4, JP2017.

**TABLE 4:** Estimated epistatic effects (AA) and environmental (AAE) interaction of QTLs for soybean 100-seed weight across all environments.

QTL_i	Chr_i	Interval_i	Pos_i	Physical position (bp)_i	QTL_j	Chr_j	Interval_j	Pos_j	Physical position (bp)_j	Epistasis – effect (AA)		Epistasis × environment effect (AAE)						
										AA	*H*^2^%	AAE1	AAE2	AAE3	AAE4	AAE5	AAE6	*H*^2^%
*qHSW-11-1_*LM6*_*	*11*	bin1245–bin1246	27.25	6,13,558,464,942,24	*qHSW–20–1_*LM6*_*	*20*	bin2175–bin2176	17.48	12725901470471	0.51**	3.46	NS	NS	NS	NS	NS	NS	0.32
*qHSW-9-1_*ZM6*_*	*9*	bin1162–bin1163	77.51	3,575,879,636,561,550	*qHSW–16–3_*ZM6*_*	*16*	bin2043–bin2044	103.81	3544126235607069	0.34**	1.38	NS	NS	NS	NS	NS	NS	0.06

Chr_i and Chr_j indicate the two sites involved in epistatic interactions; Pos indicates genetic position for each of the sites. *p < 0.05; **p < 0.01; NS, non-significant. AA indicates epistatic effects between two QTLs; those with positive values showing two-locus genotypes being the same as those in parent Linhefenqingdou and Zhengyang (or Meng 8206) have the beneficial effects, while the two-locus recombinants take the negative effects. The case of negative values is the opposite. H^2^ indicates phenotypic variation explained by epistatic effects. AE1, FY2012; AE2, JP2012; AE3, JP2013; AE4, JP2014; AE5, YC2014; and AE6, JP2017.

### Candidate Gene Mining of the Main-Effect QTLs

**FIGURE 6:**
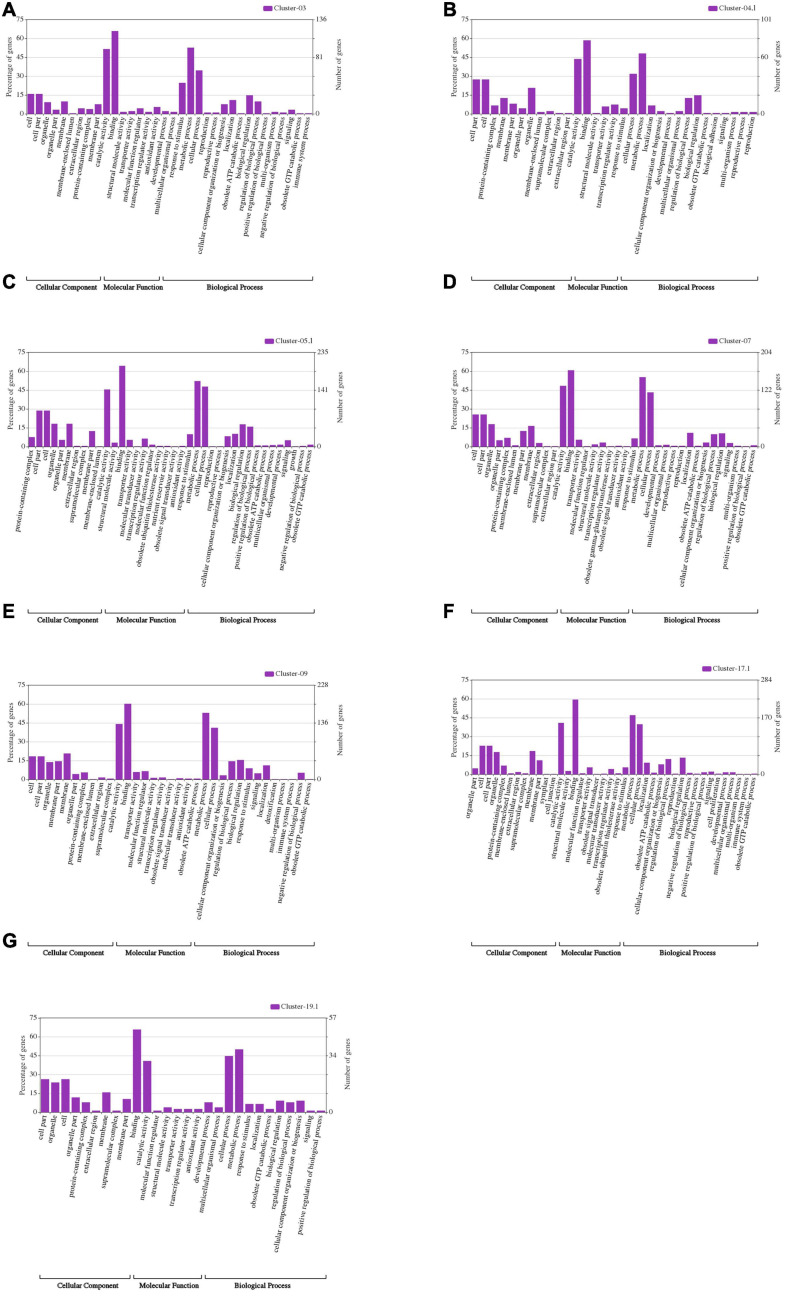
WeGO analysis of the genes located within the seven major QTL clusters: **(A)** Cluster-03; **(B)** Cluster-4.1; **(C)** Cluster-5.1; **(D)** Cluster-07; **(E)** Cluster-09; **(F)** Cluster-17.1; and **(G)** Cluster-19.1.

**FIGURE 7:**
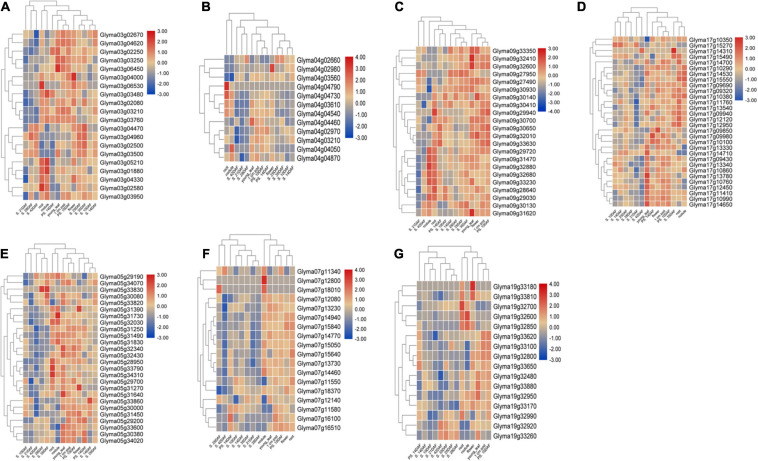
Expression profiling of 47 candidate genes across different development stages in soybean and tissues from seven QTL clusters. **(A)** Cluster-03 candidate genes, **(B)** Cluster-4.1 candidate genes, **(C)** Cluster-5.1 candidate genes, **(D)** Cluster-07 candidate genes, **(E)** Cluster-09 candidate genes, **(F)** Cluster-17.1 candidate genes, and **(G)** Cluster-19.1 candidate genes. A heat map was generated using the RNA-sequencing data retrieved from publicly available database SoyBase. PS = pod shell, DAF = days after flowering and S = Seed.

The 24 M-QTL clusters were filtered based on the richness in QTLs associated with all or some of the seed size, shape, and HSW traits. As a result, seven QTL clusters, i.e., cluster-03, 04.1, 05.1, 07, 09, 17.1, and 19.1, were used to identify candidate genes based on publicly available databases such as SoyBase and Phytozome and published papers. According to the physical intervals of the seven QTL clusters, 242, 190, 444, 367, 437, 523, and 116 genes were identified within cluster-03, 04.1, 05.1, 07, 09, 17.1, and 19, respectively, which were retrieved from the SoyBase database (see text footnote 1; Supplementary Table 11). GO enrichment analyses via AgriGO V2.0 (see text footnote 4) (Tian et al., 2017) were used to classify the model genes in each cluster. The classification was based on molecular function, biological process, and cellular components visualized on the Web-based GO (WeGO) V2.0 https://wego.genomics.cn (Ye et al., 2006). In all seven QTL clusters, high percentages of genes were related to catalytic activity, cell part, cell, cellular process, binding, and metabolic process terms, besides the response to stimulus in cluster-03 (Figure 6). These indicate the essential roles of these terms in the seed size, shape, and seed weight development in soybean. Probable candidate genes underlying these QTL clusters responsible for seed size, shape, and HSW in soybean were further predicted based on gene annotations, GO enrichment analysis, and the previously known putative biological function of the gene. Based on these, 19, 12, 26, 18, 22, 30, and 16 candidate genes were identified within the QTL clusters-03, 04.1, 05.1, 07, 09, 17.1, and 19.1, respectively (Supplementary Table 12). These genes may function directly or indirectly in regulating seed development in soybean, which regulates seed size, shape, and HSW. These genes are involved in response to brassinosteroid stimulus, regulation of cell proliferation and differentiation, regulation of transcription, secondary metabolism and signaling, storage of proteins and lipids, hormone-mediated signaling pathway, regulation of the cell cycle process, transport, ubiquitin-dependent protein catabolic process, embryonic pattern specification, and response to auxin stimulus (Table 5). However, the RNS-seq data of genes in the soybean genome (Severin et al., 2010) that is publicly available on SoyBase was used to heatmap the expression of those candidate genes in the young leaf, flower, pod, seed, root, and nodule (Figure 7 and Supplementary Table 13). From the heatmaps, 47 genes out of the identified 143 candidate genes are highly expressed during seed developmental stages and in seed-related tissues (Figure 7 and Supplementary Table 13); hence, they could be potential seed size, shape, and HSW regulatory genes.

**TABLE 5:** Candidate genes identified within the seven QTL clusters that are highly expressed in soybean seed.

QTL clusters	Gene	Start	Stop	Gene functional annotation
Cluster-03	Glyma03g01880	1668601	1674475	Seed dormancy process; protein ubiquitination; lipid storage
	Glyma03g03210	3001933	3005606	Pollen development; embryo sac egg cell differentiation; DNA-dependent
	Glyma03g03760	3581308	3584468	Maintenance of shoot apical meristem identity; cell differentiation
	Glyma03g04330	3581308	3584468	Embryo development; regulation of seed maturation
	Glyma03g04620	4798039	4801122	Regulation of meristem growth; protein deubiquitination
Cluster-04.1	Glyma04g02970	2146489	2152500	Embryo sac egg cell differentiation
	Glyma04g03210	2347024	2349849	Fatty acid beta-oxidation; response to auxin stimulus; ovule development
	Glyma04g03610	2630227	2632308	Brassinosteroid mediated signaling pathway; seed development; ovule development
	Glyma04g04460	3305860	3308715	Response to cytokinin stimulus; response to brassinosteroid stimulus; seed development
	Glyma04g04540	3395831	3397238	Response to ethylene stimulus; seed dormancy process; floral organ morphogenesis
	Glyma04g04870	3628743	3634478	Embryo development ending in seed dormancy
Cluster-05.1	Glyma05g28950	34669156	34678593	Nucleotide biosynthetic process; embryo development ending in seed dormancy
	Glyma05g29700	35236284	35242029	Brassinosteroid biosynthetic process; starch biosynthetic process
	Glyma05g30380	35754306	35755603	Embryo development; protein ubiquitination; lipid storage; anther development
	Glyma05g31450	36578952	36583516	Post-embryonic development
	Glyma05g31490	36611301	36615160	Embryo development ending in seed dormancy
	Glyma05g31830	36870586	36873840	Ubiquitin-dependent protein catabolic process
	Glyma05g32030	37026301	37031440	Ubiquitin-dependent protein catabolic process; multicellular organismal development
	Glyma05g33790	38337126	38341410	Phosphatidylcholine biosynthetic process; metabolic process
	Glyma05g34070	38511154	38513219	Cellular response to abscisic acid stimulus
Cluster-07	Glyma07g13230	11764552	11784123	Embryo sac egg cell differentiation; protein ubiquitination; lipid storage
	Glyma07g13730	12749034	12753558	Embryo development; positive regulation of gene expression
	Glyma07g14460	13903037	13906228	Embryo development ending in seed dormancy
	Glyma07g15050	14900705	14909235	Seed dormancy process; regulation of cell cycle process
	Glyma07g15640	15378798	15384642	Response to hormone stimulus and auxin stimulus; response to brassinosteroid stimulus
	Glyma07g15840	15528948	15544150	Ubiquitin-dependent protein catabolic process; regulation of lipid catabolic process
Cluster-09	Glyma09g28640	35573357	35579018	Embryo development ending in seed dormancy; cellular response to abscisic acid stimulus
	Glyma09g29030	35989729	35993075	Ubiquitin-dependent protein catabolic process; fatty acid beta-oxidation
	Glyma09g29720	36540972	36548174	Response to auxin stimulus; auxin metabolic process
	Glyma09g30130	37014420	37023261	Protein import into nucleus; embryo sac egg cell differentiation
	Glyma09g30650	37426876	37433118	Phosphatidylcholine biosynthetic process; metabolic process; pollen development
	Glyma09g31620	38298193	38307446	Response to abscisic acid stimulus; embryo development
	Glyma09g32600	39100482	39107332	Translational elongation; embryo development ending in seed dormancy
	Glyma09g32680	39173955	39183935	Regulation of protein phosphorylation
	Glyma09g33630	40063507	40067999	Response to auxin stimulus; seed dormancy process
Cluster-17.1	Glyma17g09320	6889969	6894069	Seed maturation; histone deacetylation; response to abscisic acid stimulus
	Glyma17g09690	7171761	7186015	Seed maturation; protein ubiquitination; lipid storage
	Glyma17g10290	7707775	7711360	Pollen tube growth; seed dormancy process; ovule development
	Glyma17g10380	7768561	7778131	Ubiquitin-dependent protein catabolic process
	Glyma17g10990	8262700	8267178	Carbohydrate metabolic process
	Glyma17g11410	8557013	8563158	Regulation of embryo sac egg cell differentiation
	Glyma17g12950	9873806	9891306	Protein folding; embryo development response to starvation
	Glyma17g15490	12218497	12226562	Ubiquitin-dependent protein catabolic process
	Glyma17g15550	12302621	12306143	N-terminal protein myristoylation; pollen development; pollen tube growth
Cluster-19.1	Glyma19g32990	40666918	40669847	Glucose catabolic process; response to auxin stimulus
	Glyma19g33620	41194146	41196743	Maltose metabolic process; starch biosynthetic process; glucosinolate biosynthetic process
	Glyma19g33650	41237306	41242657	Abscisic acid biosynthetic process; plant-type cell wall modification; pollen tube growth

## Discussion

The present study has implemented high-density genetic maps constructed from two-related RIL populations LM6 and ZM6 comprising 2,267 and 2,601 bin markers, respectively (Li et al., 2017), to validate QTLs associated with seed size, shape, and weight. To minimize the environmental errors, the two RIL populations were evaluated in four environments. The transgressive segregation and continuous variations observed in the two populations in all studied phenotypic traits facilitate the identification of a high number of both major and minor effect QTLs including some novel QTLs associated with all studied traits (Teng et al., 2009; Xu et al., 2011; Zhang et al., 2018). All measured and calculated traits in both populations were significantly (*P* < 0.01) influenced by genotype (G), environment (E), and their interactions (G × E), suggesting that the seed size, shape, and weight traits are not only governed by both genetic and environment; however, there was an effect of the G × E interaction as well (Sun et al., 2012; Hu et al., 2013; Liang et al., 2016). This explains the observed high *h*^2^ (99.04%) and accordingly deduces that these traits are amenable to manipulation by selection without the help of molecular markers. Except for SL, SW, and ST that exhibited a highly significant correlation between each other and with HSW, our data showed that seed size, shape, and weight traits are not correlated, which is favorable when breeding for a round type with smaller or bigger seed size (Cober et al., 1997; Salas et al., 2006).

For validation of identified QTLs, a comparative QTL analysis using the CIM QTL mapping approach with the SoyBase database identified 69, 82, and 29 novel QTLs for seed size, shape, and HSW, respectively, indicating the distinct genetic architecture of the LM6 and ZM6 populations. These novel QTLs together explain over 88.00% of phenotypic variance for seed size, shape, and weight, signifying their potential value for improving soybean cultivars. Besides, the identification of novel QTLs in the present study suggests that more germplasms are required for unraveling the complex genetic basis for seed size and shape traits in soybean. Among these novel QTLs, eight novel major QTLs associated with HSW where their physical intervals did not overlap with any of the previously reported HSW QTLs, suggesting them as potential loci for HSW and major QTLs for future fine mapping to delimit the physical interval. Numerous QTLs associated with SW, HSW, SLW, and SLT identified in this study are co-localized with previously reported corresponding QTLs (Salas et al., 2006; Li et al., 2010; Moongkanna et al., 2011; Hu et al., 2013; Jun et al., 2014; Fang et al., 2017; Hacisalihoglu et al., 2018; Hina et al., 2020). Our study identified for the first time 13 major QTLs (*R*^2^ > 10%) related to FI; thus, we considered them as novel QTLs. Besides, Chr01 and Chr03 harbored four and three FI QTLs, suggesting crucial roles of Chr 01 and 03 in controlling the inheritance of seed FI in soybean. The positive alleles for seed size, shape, and HSW traits were inherited from both parents of the two RIL populations. Therefore, it is likely that not only the higher seed size and heavyweight parent (Linhefenqingdou or Zhengyang) contributed favorable alleles but also the lighter seed weight parent (M8206) might play a role (Cao et al., 2019; Hina et al., 2020).

Mapping of QTLs associated with seed size, shape, and weight-related traits using the MCIM approach was performed to (i) dissect the additive effect QTLs and Q × E interactions, which is essential for selecting the most compatible varieties adapted to particular environments, and (ii) further validate the QTLs identified by the CIM approach. The MCIM approach identified 18 QTLs for seed sizes, shapes, and weight traits that are co-localized in the same physical interval of the CIM-mapped QTLs. Therefore, these QTLs could also be stable QTLs for further fine mapping and map-based cloning to uncover the genetic control and mechanisms of seed size, shape, and weight traits in soybean, and molecular markers tightly linked to these QTLs could be used for MAS.

Dissecting the epistatic and QTL × environment effects are crucial for understanding the genetic mechanisms that contributed to the phenotypic variations of complex traits (Kaushik et al., 2007). Disregarding intergenic interactions will lead to the overestimation of individual QTL effects, and the underestimation of genetic variance resulting in a large drop in the genetic response to MAS especially in late generations (Nyquist and Baker, 1991; Zhang et al., 2004). The identified 40 pairwise digenic epistatic QTLs for seed size, shape, and weight-related traits in the present study could be considered as modifying genes that do not exhibit only additive effects but could affect the expression of seed size, shape, and weight-related genes through epistatic interactions. Similar results for the epistatic interaction of seed size, shape, and weight QTLs have been also previously reported by Xin et al. (2016) and Zhang et al. (2018). The appearance of epistatic interactions for a specific trait makes selection difficult. Noteworthily, all main-effect QTLs detected in our study had no epistatic effect, which raises the heritability of the trait guiding to easier selection.

Genomic regions were identified as QTL clusters based on the presence of several QTLs related to all or some of the seed size, shape, and HSW traits. Accordingly, 24 QTL clusters were identified on 17 chromosomes each containing three or more QTLs related to seed size, shape, and HSW traits. These QTL clusters have not been previously reported, which enhances the developing knowledge of the genetic control of these traits. The co-localization of QTLs for seed size, shape, and HSW and how they have exceptionally corresponded support the highly significant correlation with each other (Cai and Morishima, 2002) (Supplementary Table 10). Besides, the occurrence of the QTL clustering could signify a linkage of QTLs/genes or outcome from the multiple effects of one QTL in the same genomic region (Wang et al., 2006; Cao et al., 2017; Liu et al., 2017). The QTL clusters reveal that the QTL linkage/gathering could make the enhancement of seed size and shape easier than single QTLs (Hina et al., 2020). Significant positive correlations of soybean seed protein and oil contents and seed yield with seed size and seed shape have been shown; therefore, these traits are directly associated with seed size and shape in soybean (Qi et al., 2011; Hacisalihoglu and Settles, 2017; Wu et al., 2018). This notion would explain the co-localization of QTLs associated with seed protein and oil contents in the genomic regions of several QTL (Panthee et al., 2005; Salas et al., 2006; Vieira et al., 2006; Moongkanna et al., 2011; Yang et al., 2011). The position of the first flower and the number of days to flowering have large effects on seed number per plant in soybean (Tasma et al., 2001; Yamanaka et al., 2001; Khan et al., 2008), which affects seed size and HSW indicating the existence of common genetic factors for these traits. QTLs associated with the position of the first flower identified previously (Tasma et al., 2001; Han et al., 2012) are located to the genomic region of clusters 16.1, 19.2, and 20 (Hyten et al., 2004). The extensive analysis of QTL clusters in our study suggests that breeding programs aiming to improve seed size, shape, and weight with enhanced quality should focus on QTL clustering and select QTLs within these regions. Besides, the existence of QTL clusters provides evidence that some traits-related genes are more densely concentrated in specific genomic regions of crop genomes than others (Fang et al., 2017).

Identification of candidate genes underlying QTL regions is of great interest for breeding programs (Abou-Elwafa, 2018; Abou-Elwafa and Shehzad, 2018). A bioinformatics pipeline implementing genomic sequences of identified QTL clusters was employed to identify candidate genes. The pipeline comprises three complementary steps, i.e., (1) retrieving candidate genes from the SoyBase database, (2) visualizing the molecular function of candidate genes by GO enrichment analyses and gene classification, and (3) implying candidate genes in seed size, shape, and weight based on their expression profiles. Accordingly, 47 genes were considered as potential candidates. Most of the identified candidate genes are related to the terms of catalytic activity, cell part, cell, cellular process, and binding and metabolic process as indicated by GO enrichment and gene classification analyses. These terms have functions related/involved in seed development, which influence the size, shape, and weight of seeds (Mao et al., 2010; Li and Li, 2014). For example, the *Glyma07g14460* gene underlying QTL cluster-7 belongs to the oxygenase (CYP51G1) protein class, which has been confirmed to regulate seed size in soybean (Zhao et al., 2016). Furthermore, 10 candidate genes were identified as a regulator of ubiquitin-dependent protein catabolic process, RING-type E3 ubiquitin ligases, and lipid catabolic process (Table 5). Several components of the ubiquitin pathway such as the ubiquitin-activating enzyme (E1), ubiquitin-conjugating enzyme (E2), and ubiquitin protein ligase (E3) have been reported to play important roles in regulation seed and organ size (Li and Li, 2014). Similarly, 16 candidate genes have functions in pollen tube development, embryo sac egg cell differentiation, post-embryonic development, regulation of seed maturation, positive regulation of gene expression, regulation of cell cycle process, ovule development, anther development, seed dormancy process, and seed maturation (Table 5), and hence they are likely to participate in regulating seed size, shape, and weight in plants, including soybean (Meng et al., 2016). Ten candidate genes are involved in response to auxin stimulus, response to ethylene stimulus, and abscisic acid biosynthetic process which are known to be implicated in promoting seed size and weight in Arabidopsis (Table 5) (Xie et al., 2014). Six genes play functions in the glucose catabolic process, phosphatidylcholine biosynthetic process, carbohydrate metabolic process, maltose metabolic process, and starch biosynthetic process and are implicated in the partitioning and translocation of photoassimilates and grain filling in rice (Table 5) (Chen J. et al., 2020; Zhang et al., 2020).

## Conclusion

QTLs associated with seed size, shape, and weight in soybean were identified and validated using two mapping approaches in two populations across multiple environments. This is the first comprehensive investigation of the identification and validation of QTLs for the FI as a seed shape trait in soybean. Employing a bioinformatics pipeline identified candidate genes behind genomic regions harboring major and stable QTL clusters underlying the inheritance of seed size, shape, and weight. The implemented bioinformatics pipeline delimits the number of the identified candidate genes to 47-gene genomic regions involved directly or indirectly in seed size, shape, and weight. These genes are highly expressed in seed-related tissues and nodules, indicating that they may be involved in regulating these traits in soybean. Furthermore, some of the potential 47 candidate genes have been included in our ongoing projects for functional validation to confirm their effect on seed size, shape, and weight. Our study provides detailed information for genetic bases of the studied traits and candidate genes that could be efficiently implemented by soybean breeders for fine mapping and gene cloning and for MAS targeted at improving seed size, shape, and weight.

## Data Availability Statement

The datasets presented in this study can be found in online repositories. The names of the repository/repositories and accession number(s) can be found in the article/Supplementary Material.

## Author Contributions

TZ designed the project. ME performed the experiments and drafted the manuscript. ME, BK, SS, SL, YC, MA, and AH analyzed the data. TZ and SA-E revised the manuscript. All authors have read and agreed to the published version of the manuscript.

## Conflict of Interest

The authors declare that the research was conducted in the absence of any commercial or financial relationships that could be construed as a potential conflict of interest.

## Footnotes

1 www.soybase.org

2 http://www.spss.com

3 http://phytozome.jgi.doe.gov

4 http://systemsbiology.cau.edu.cn

## Supplementary Material

Supplementary Figure 1 Seed size, shape, and seed weight traits related QTLs mapped using CIM approach in the two soybean RIL populations LM6 and ZM6 across multiple environments indicated with E1, FY2012; E2, JP2012; E3, JP2013; E4, JP2014; E5, YC2014; E6, JP2017 respectively, in addition to the combined environment (CE). **(a)** Seed length associated QTLs, **(b)** seed width associated QTLs, **(c)** seed thickness associated QTLs, **(d)** seed length/width associated QTLs, **(e)** seed length/thickness associated QTLs, **(f)** seed width/thickness associated QTLs, **(g)** FI associated QTLs, and **(h)** QTLs associated with HSW. The LOD threshold (2.5) is indicated by a green line. The *X* and *Y*-axis represent chromosomes and LOD score, respectively.

Supplementary Figure 2 Diagram showing Chromosomal locations of the identified 24 QTL clusters on 17 different chromosomes, i.e., Chr 01, 03, 04, 05, 06, 07, 08, 09, 10, 11, 13, 14, 15, 16, 17, 19, and 20 in LM6 and ZM6 RIL populations for SL, SW, ST, SLW, SLT, SWT, FI, and HSW traits under multiple environments.

Supplementary Table 1 Distribution of SNPs, recombination bins and markers mapped on soybean chromosomes/linkage groups. Bin-map (RAD-sequencing).

Supplementary Table 2 Descriptive statistics, variance components, and broad-sense heritability (*h*^2^) of seed shape and size traits evaluated in two recombinant inbred lines (RILs) LM6 and ZM6. The RILs and their parents were grown under different environments. ^∗^ and ^∗∗^ represent significance at 5 and 1%, respectively.

Supplementary Table 3 Descriptive statistics, variance components, and broad-sense heritability (h^2^) for 100-Seed weight trait evaluated in two recombinant inbred lines (RILs) LM6 and ZM6. The RILs and their parents were grown under different environments. ^∗^ and ^∗∗^ represent significance at 5 and 1%, respectively.

Supplementary Table 4 Combined analysis of variance (ANOVA) for Seed shape and size trait (SL, SW, ST, SLW, SLT, SWT, FI) in ZM6 and LM6 RIL Populations across four different environments (2012FY, 2012JP, 2014JP and 2017JP).

Supplementary Table 5 Combined analysis of variance (ANOVA) for 100-Seed weight trait in ZM6 and LM6 RIL Populations across six different environments (2012FY, 2012JP, 2013JP, 2014JP, 2014YC, and 2017JP).

Supplementary Table 6 Correlation analysis among seven Seed size/Shape traits (SL, SW, ST, FI, SLT, SLW, SWT), and 100-seed weight (HSW). ^∗^ and ^∗∗^ represent significance at 5 and 1%, respectively.

Supplementary Table 7 Main-effect quantitative trait loci (M-QTLs) identified for three seed-size traits (SL, SW, and ST in ZM6 and LM6 recombinant inbred line (RIL) populations across multiple environments and combined environment.

Supplementary Table 8 Main-effect quantitative trait loci (M-QTLs) identified for four seed-shape traits [Seed length/width ratio (SLW), Seed length thickness ratio (SLT), Seed width thickness ratio (SWT), and Flatness index (FI)] in ZM6 and LM6 recombinant inbred line (RIL) populations across multiple environments and combined environment.

Supplementary Table 9 Main-effect quantitative trait loci (M-QTLs) identified for 100-Seed weight in ZM6 and LM6 recombinant inbred line (RIL) populations across multiple environments and combined environment.

Supplementary Table 10 Twenty-Four QTL clusters detected in LM6 and ZM6 RIL populations across multiple environments.

Supplementary Table 11 Model genes within Cluster-03, Cluster-04.1, Cluster-05.1, Cluster-07, Cluster-09, Cluster-17.1, and Cluster-19.1 regions.

Supplementary Table 12 Predicted candidate genes within Cluster-03, Cluster-04.1, Cluster-05.1, Cluster-07, Cluster-09, Cluster-17.1, and Cluster-19.1 regions in both RIL Populations (LM6 and ZM6) based on known functional annotation (highlighted lines show candidate genes).

Supplementary Table 13 Candidate genes within seven QTL clusters and their relative expression data retrieved from RNA-seq data available in SoyBase (DAF = days after flowering) (highlighted lines show candidate genes).
