# Supplementary figures and images for: Identification and Validation of Major QTLs, Epistatic Interactions, and Candidate Genes for Soybean Seed Shape and Weight Using Two Related RIL Populations
Source: Front Genet. 2021 May 28;12:666440. doi: 10.3389/fgene.2021.666440 (PMC8195344; doi:10.3389/fgene.2021.666440)

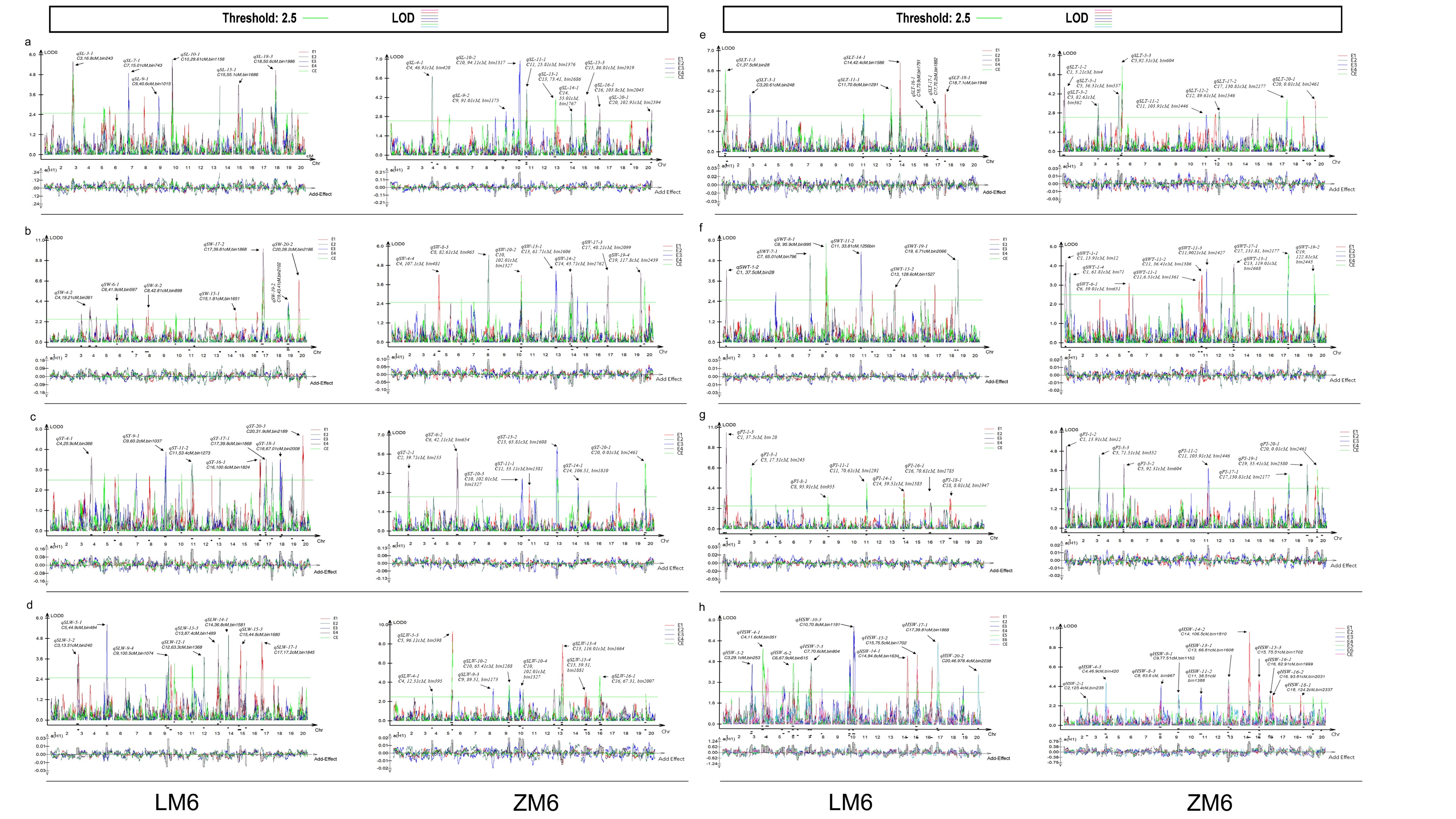

Supplement: Supplementary Figure 1 — Seed size, shape, and seed weight traits related QTLs mapped using CIM approach in the two soybean RIL populations LM6 and ZM6 across multiple environments indicated with E1, FY2012; E2, JP2012; E3, JP2013; E4, JP2014; E5, YC2014; E6, JP2017 respectively, in addition to the combined environment (CE). (a) Seed length associated QTLs, (b) seed width associated QTLs, (c) seed thickness associated QTLs, (d) seed length/width associated QTLs, (e) seed length/thickness associated QTLs, (f) seed width/thickness associated QTLs, (g) FI associated QTLs, and (h) QTLs associated with HSW. The LOD threshold (2.5) is indicated by a green line. The X and Y-axis represent chromosomes and LOD score, respectively. [file Image_1.jpg]

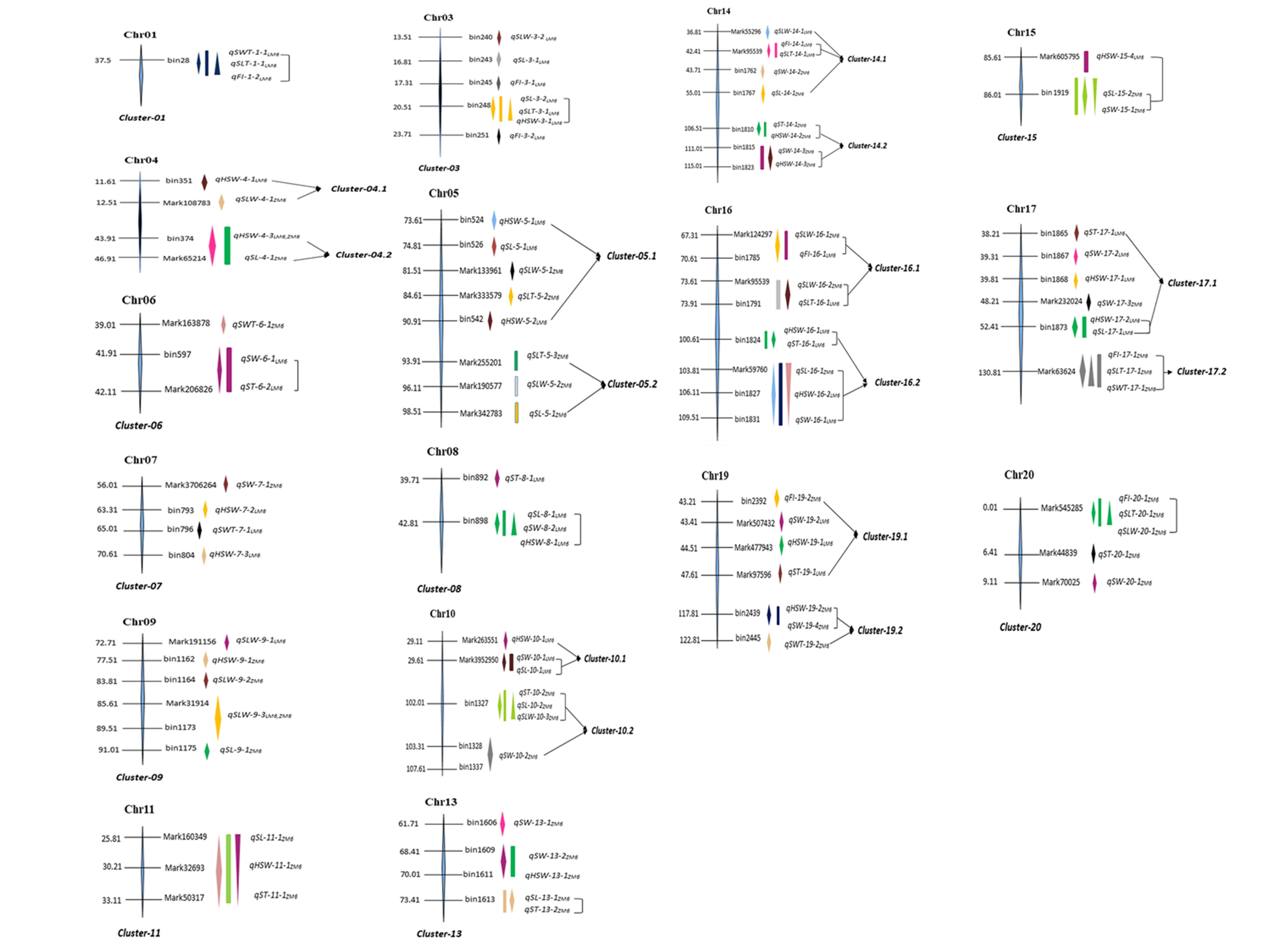

Supplement: Supplementary Figure 2 — Diagram showing Chromosomal locations of the identified 24 QTL clusters on 17 different chromosomes, i.e., Chr 01, 03, 04, 05, 06, 07, 08, 09, 10, 11, 13, 14, 15, 16, 17, 19, and 20 in LM6 and ZM6 RIL populations for SL, SW, ST, SLW, SLT, SWT, FI, and HSW traits under multiple environments. [file Image_2.jpg]
